# GWAS analysis of Fusarium head blight resistance in a Nordic-Baltic spring wheat panel

**DOI:** 10.3389/fpls.2025.1604296

**Published:** 2025-07-23

**Authors:** Shayan Syed, Andrius Aleliūnas, Rita Armonienė, Gintaras Brazauskas, Andrii Gorash

**Affiliations:** ^1^ Department of Cereal Breeding, Institute of Agriculture, Lithuanian Research Centre for Agriculture and Forestry, Akademija, Lithuania; ^2^ Laboratory of Genetics and Physiology, Institute of Agriculture, Lithuanian Research Centre for Agriculture and Forestry, Akademija, Lithuania

**Keywords:** FHB, genome wide association analysis, QTL, wheat resistance, candidate genes, gene pyramiding

## Abstract

Genetic improvement of wheat resistance to the devastating disease Fusarium head blight (FHB) is the most effective strategy to prevent economic, health, and food safety issues, and is also an environmentally friendly approach for disease control. However, wheat breeding for FHB resistance is hampered by complex resistance, which is controlled by multiple loci with minor effects and limited availability of resistance sources. Globally, sources of FHB resistance primarily stem from Asian wheat; however, excellent resistance has also been noted in European spring wheat cultivars and breeding lines. The success of breeding for the improvement of wheat resistance to FHB relies on the availability of a genetic pool that is adapted to local environments, possesses desirable agronomic traits, and includes a sufficient number of effective QTL for wheat resistance to FHB. A genome-wide association study (GWAS) was performed using a panel of 332 spring wheat genotypes including 181 from Baltic, Nordic countries (65), Central and Western Europe (76) and exotic genotypes (10), employing a 25 K single nucleotide polymorphism (SNP) array. The objectives of this study were to identify SNPs significantly associated with wheat resistance, determine QTL with approximate regions, and identify candidate genes within these QTL by exploring a panel of wheat genotypes adapted to the Baltic and Nordic countries. A total of 65 significant marker-trait associations (MTAs) with FHB resistance were identified using GWAS. Resistance loci were distributed across 15 wheat chromosomes and three genomes. Furthermore, 55 QTL were identified, 10 of which had phenotypic variation explained (*R^2^*) values above 10%. *QFHB-2AL.1* and *QFHB-2BL.1* were stably detected in 11 trials. An overall total of 52 candidate genes was identified by analyzing QTL regions in combination with published transcriptome data. This study demonstrated that a substantial number of QTL can be found in European spring wheat germplasm. Pyramiding of major effects along with small-effect QTL resulted in a positive additive effect on wheat resistance. Elite breeding lines with multiple resistance alleles were identified and could be used as valuable sources in wheat breeding for FHB resistance.

## 1 Introduction

Bread wheat (*Triticum aestivum* L.) is a principal cereal crop, with an annual production of 760 million tons covering 219 million hectares (avg. of 10 years), contributing approximately 20% of the daily caloric intake of humans globally (Alisaac and Mahlein, 2023). Furthermore, it is predicted that a 60% increase in productivity is required to meet the food demands of the global population, which is expected to reach 9.6 billion by 2050 (Bahar et al., 2020). To fulfill this demand, there is a need to increase the production of wheat crops by reducing the gap between the actual and potential grain yields, which can be achieved by improving disease resistance.

Fusarium head blight (FHB), also known as scab, is caused by at least nineteen *Fusarium* species, of which *Fusarium culmorum, F. graminearum*, and *F. avenaceum* are the most aggressive and widespread species. It is a devastating disease that not only drastically lowers wheat yield but also degrades end-use quality by toxicating seeds through mycotoxin accumulation. It also has a significant negative impact on human health, animal health, and productivity, as well as on domestic and international trade (Pestka, 2010; Pitt and Miller, 2017; Wu and Mitchell, 2016). Due to the health risks caused by the two main mycotoxins, deoxynivalenol (DON) and zearalenone (ZON), the improvement of resistance to FHB has become a top priority among all cereal diseases in the European Union (Miedaner et al., 2024). Furthermore, severe FHB epidemics have been reported in wheat-producing countries worldwide (Bai et al., 2018a; Bai and Shaner, 2004; Buerstmayr et al., 2009; Dubin, 1997; McMullen et al., 2012). Modifications in agricultural practices (such as zero tillage and simplified crop rotation), together with increased global warming, provide FHB pathogens with more favorable environmental conditions to flourish, even in areas where FHB has not been previously observed (Alisaac and Mahlein, 2023; Bai et al., 2018b; Dill-Macky and Jones, 2000; Juroszek and von Tiedemann, 2015). A substantial shift in Fusarium head blight area has been observed in northern European regions, where the impact of the disease was previously negligible (Miedaner et al., 2024; Miedaner and Juroszek, 2021). The presence of *Fusarium* spp. in Danish and Norwegian crops was reported in the late 20^th^ and early 21^st^ centuries (Andersen et al., 1996; Kosiak et al., 2003; Nielsen et al., 2011; Sundheim et al., 2013; Thrane, 2000). Moreover, the first detection of *Fusarium* spp. in Finland was published in 2019, indicating the emergence of the pathogen in an area where it had not been previously identified (Gagkaeva and Yli-Mattila, 2020; Hautsalo, 2020). Similarly, in Lithuania, FHB was not a notable threat until 2003; however, it has evolved into a considerable concern since 2012 (Mačkinaitė et al., 2006; Sakalauskas et al., 2014; Suproniene et al., 2016).

A comprehensive approach is required to control this disease, which encompasses various strategies, such as agricultural crop management practices, application of fungicides, and enhancement of plant resistance. However, the use of fungicides not only increases the risk of environmental contamination, but also raises the cost of production (McMullen et al., 2012). Conclusively, breeding wheat for resistance to FHB is the most crucial approach because of its greater effectiveness, durability, economic viability, and environmental safety (Bai and Shaner, 2004; Buerstmayr et al., 2014; Buerstmayr et al., 2009; Ghimire et al., 2022; McMullen et al., 2012; Steiner et al., 2017). To date, more than 600 quantitative trait loci (QTL) have been identified so far which are related to FHB resistance (Zheng et al., 2021). Gaire et al. (2021) suggested that pyramiding QTL from various genetic sources is an efficient strategy for improving FHB resistance in wheat cultivars (Gaire et al., 2021). However, the objective is complicated by the quantitative inheritance of resistance to FHB, which involves numerous QTL with minor effects, whereas major and stable QTL are easier to apply in breeding programs than small-effect QTL (Hu et al., 2020). The marker-assisted selection (MAS) technique provides effective results when the trait is controlled by a few major QTL, whereas it is not sufficiently effective when a trait is controlled by multiple small-effect QTL. The transfer of a single QTL, even with a major effect, such as *Fhb1*, does not yield satisfactory outcomes (Mesterhazy, 2020). However, advanced genetic approaches, such as genome-wide association studies (GWAS) that leverage linkage disequilibrium (LD) and employ high-density SNP markers, allow the detection of a large number of significant marker-trait associations for FHB resistance (Hu et al., 2020; Verges et al., 2021). LD mapping or association mapping enables the identification of associations between single nucleotide polymorphisms (SNPs) and complex phenotypic traits in a diverse germplasm population, essentially increasing mapping resolution over standard mapping populations (Flint-Garcia et al., 2003; Myles et al., 2009; Sahoo et al., 2022; Scherer and Christensen, 2016). LD analysis narrows down the genomic regions associated with phenotypic traits, hence pinpointing candidate genes located within these regions (Ma et al., 2022; Yan et al., 2020), such as FHB disease resistance genes. Moreover, the identified SNP markers can be applied successfully through MAS, if several major QTL are detected or can be incorporated into genomic selection (GS). In addition, GS is a powerful tool for complex traits, such as resistance to FHB, which enables the incorporation of multiple small-effect QTL, reduces the cost of phenotyping, and accelerates the breeding process (Arruda et al., 2016).

Fusarium head blight is caused by a hemibiotrophic pathogen that penetrates the plant as biotroph and then shifts to the necrotrophic phase (Trail, 2009), approximately 48 h after inoculation (Seifi et al., 2023). According to the general concept, wheat resistance to FHB encompasses five different types or components of resistance: resistance to penetration (Type I), spreading within plant tissue (Type II), kernel resistance (Type III), resistance to toxin accumulation (Type IV), and tolerance to yield reduction (Type V). Moreover, overall resistance, which includes both Types I and II, has come into focus as an additional Type of resistance (Mesterházy et al., 2018, Mesterházy et al., 2015; Zwart et al., 2008a). Although these types of resistance do not function as isolated systems, some genes overlap, expressed proteins interact, and activated pathways are interrelated (Mesterhazy, 2024; Sirangelo, 2024). The complete molecular mechanisms of all these types of resistance, and their interactions remain largely unknown. However, according to recent transcriptomic studies, wheat resistance to FHB is controlled by thousands of genes and is achieved through the downregulation and upregulation of gene expression (Buerstmayr et al., 2021).

Basal resistance (Type I), which provides the first layer of defense, involves the upregulation of genes that are involved in cell wall reinforcement (activation of structural proteins such as glycine-rich proteins and hydroxyproline-rich proteins (Kang and Buchenauer, 2003; Kikot et al., 2009; Soni et al., 2020; Walter et al., 2010) and secondary metabolite production, such as terpenoids (Singh and Sharma, 2015). Type II of resistance, the spread of pathogen through the rachis is associated with the downregulation of reactive oxygen species (ROS), programmed cell death (PCD) and upregulation of ROS scavenging. ROS activation induces the expression of trichothecene biosynthetic genes (TRI), which produce DON mycotoxins in fungi and promote pathogen spread. Due to the pathogen’s ability to switch from the biotrophic to the necrotrophic phase, the activation of PCD pathways in plants promotes the necrotrophic phase of the fungus, while the suppression of PCD helps to restrict the growth of the fungus. Recent studies have demonstrated that susceptible wheat genotypes exhibit excessive expression of PCD-related genes, whereas the expression of such genes is limited in resistant genotypes. Type III wheat resistance is mainly associated with the upregulation of genes encoding UDP-glycosyltransferases (UGTs), glutathione S-transferases (GSTs), and ATP-binding cassette (ABC) transporter expression, which helps in the detoxification of DON mycotoxins (He et al., 2020; Sirangelo, 2024; Walter et al., 2015; Zhao et al., 2018). Additionally, some genes encode hormones that participate in the regulation of signaling pathways (jasmonic acid and salicylic acid) or in secondary metabolite production (terpenoids) (Buerstmayr et al., 2021; Seifi et al., 2023). Previous studies have demonstrated that *Fhb1* and *Qfhs.ifa-5A* contain genes that are largely involved in secondary cell wall biogenesis and terpene metabolism (Buerstmayr et al., 2021). Exploring candidate genes within the QTL of resistance and their putative roles in defense might facilitate the proper choice of QTL pairing/accumulation in breeding strategies to develop improved and durable wheat resistance to FHB.

Nevertheless, intensive global efforts have been initiated to enhance FHB resistance in bread wheat. No substantial genetic improvement in wheat resistance has been found among registered wheat cultivars over the last 20 years (Mesterházy et al., 2018; Miedaner et al., 2024). Improving quantitative traits is a challenging task that requires several cycles of breeding to accumulate the associated alleles/QTL in one genotype. Furthermore, the associated QTL may have a linkage drag with FHB resistance. All well-known QTL with major effects, such as *Fhb1*, *Fhb4*, and *Fhb5*, are derived from exotic germplasm, and in addition to conferring resistance, also impart undesirable agronomic characteristics. The task is more feasible when resistance to FHB is identified in wheat cultivars or breeding lines adapted for growing in areas of Europe, as such materials possess resistance QTL and do not have a linkage with undesirable traits. Therefore, the identification of resistance within breeding populations derived from locally adapted germplasms is paramount for improving FHB resistance (Zwart et al., 2008b).

The objective of the present study was to perform a GWAS in a panel of 332 wheat genotypes using phenotypic data from multiple trials to identify significantly associated SNP markers, QTL, and candidate genes related to FHB resistance. This collection comprises the local breeding lines and adapted cultivars for the Nordic and Baltic regions, which possess high yield performance and/or other valuable agronomic traits, such as high grain quality, resistance to diseases, drought stress, and other important agronomic traits. Dissection of resistance and identification of QTL associated with FHB resistance in this wheat collection would provide information about the genetic architecture of FHB resistance in modern cultivars adapted to the Baltic and Nordic regions and may assist in the development of new spring wheat cultivars with improved FHB resistance and adapted to the region.

## 2 Materials and methods

### 2.1 Experimental materials and trials

In this study, 335 spring wheat genotypes (including 325 European and 10 exotic genotypes from CIMMYT, China and Africa) were used to assess FHB resistance under field and controlled conditions. Initially, a set of 300 genotypes (breeding lines and varieties) from different origins, such as Norway, Estonia, Latvia, and Lithuania were genotyped and phenotyped for other agronomic traits, except for FHB resistance (Aleliūnas et al., 2024; Lin et al., 2024). Furthermore, for this study, we included 30 advanced Lithuanian breeding lines, the Slovenian cultivar PS Perlička, and the Chinese resistant landrace Wangshuibai that showed better performance in FHB resistance during one or two field inoculation cycles to the given set. During genetic quality control, three genotypes were excluded. Finally, the set comprised 332 genotypes, of which 65 originated from Nordic countries, 181 from Baltic countries, 76 from Central and Western Europe, and 10 were exotic (foreign origin). There were well-known check cultivars with high resistance and susceptibility to FHB in the group of foreign origin group. For instance, the well-known Chinese highly resistant cultivar Sumai 3 and landrace Wangshuibai were used as resistance checks, while the South African cultivar Gamenya was used as a check for susceptibility. Other exotic genotypes, such as CIMMYT FHB resistant lines SHA3/CBRD and MILAN/SHA7 and the Chinese FHB resistant line N894037, also have well-known resistance and were included as references of resistance. The field experiments were carried out at the Institute of Agriculture, Lithuanian Research Centre for Agriculture and Forestry (LAMMC) in 2022 and 2023. Genotypes were grown in Alpha experimental design in a 1.5 m^2^ plot with two replicates. Spray inoculation method was used in the field and greenhouse. The spikes were covered with plastic bags for 48 h to maintain moisture and were later assessed for type I resistance to FHB. For the spawn grain inoculation method, the genotypes were grown in pots and kept in a nursery where sprinklers were installed to create a humid environment for the plants. To inoculate plants, spawn grains were applied at a rate of 30 g/m^2^ at the stem elongation time, early booting stage, and at the end of the booting stage (Tekle et al., 2018). The same technique was followed for the point inoculation method in the greenhouse, where plants were grown in pots at 20-23°C temperature. When the plants reached the anthesis stage, the inoculum was injected into the central florets through glumes to provide mock disease pressure for each genotype to evaluate them for type II resistance. To conserve moisture, the spikes were covered with plastic bags for two days. The details of the trial design, preparation and concentration of inoculums, inoculation techniques and meteorological conditions were described in detail previously (Syed et al., 2024).

### 2.2 Analysis of phenotypic data

For phenotypic data, disease severity, FHB index and FHB incidence were recorded.

#### 2.2.1 Evaluation of overall resistance

To evaluate disease severity, genotypes were spray inoculated in fields and greenhouse. After two weeks of inoculation, genotypes were visually assessed for severity. The infected spikes were evaluated visually by using 0-100% scale. Those genotypes having symptoms in one-fourth part of the spike considered 25% severity, if half spike have symptoms of FHB, then 50% and so on. The similar procedure was followed for evaluating severity in the spawn grain method. To evaluate FHB severity, again a 0-100% scale range was used (Stack and McMullen, 1998).

For calculating FHB index, disease incidence and disease severity data were used to calculate index percentage:


$$FHB\;Index=\frac{Disease\;incidence\;\times \;disease\;severity}{100}$$

#### 2.2.2 Evaluation of type II resistance

To assess Type II resistance we used point inoculation method under greenhouse conditions, in which diseased and healthy spikelets per spike were counted. Later, the percentage of disease severity for point inoculated genotypes was calculated by:


$$Disease\;severity=\frac{Number\;of\;infected\;spikelets}{Total\;number\;of\;spikelets/spike}\times 100$$

#### 2.2.3 Evaluation of type I resistance

For FHB incidence, genotypes were visually evaluated after seven days of inoculation under field conditions. The bunch of inoculated spikes were assessed as healthy or diseased spikes based on visual signs and symptoms of initial FHB infection. To calculate incidence, this formula was used:


$$Disease\;incidence=\frac{Diseased\;spikes}{Total\;number\;of\;spikes}\times 100$$

### 2.3 Plant genotyping, population structure

Genotyping of the 335 wheat genotypes performed using 25K SNP chip array at TraitGenetics GmbH (Germany) as described previously (Aleliūnas et al., 2024; Lin et al., 2024) under the framework of the NOBAL wheat project. Raw marker data were obtained from a previous study (Aleliūnas et al., 2024; Lin et al., 2024). Additionally, 35 genotypes which were selected as resistant in one of two cycles of pre-breeding for FHB resistance were included in this study, and genotypic data curation and quality control were performed separately. Monomorphic markers were removed from the dataset along with completely failed markers. Afterwards, the filtering was done to remove the markers with more than 20% missing data and minor allele frequency (MAF) greater than 0.05. After the filtering, a subset of 18,417 high-quality genetic markers were selected for the analysis. Overall, 332 genotypes passed quality control and were included in GWAS.

### 2.4 Statistical analysis

GWAS was performed using a Bayesian-information and Linkage-disequilibrium Iteratively Nested Keyway (BLINK) model with two the first principal components as covariates (Huang et al., 2019) in R package “GAPIT3” (Wang and Zhang, 2021). To find out the optimal number of principal components (PCs), the Elbow method was applied (**Supplementary Figure S1**). The scree plot was built using “ggplot2” and “ggrepel” R packages for visualization. The genomic Inflation Factor (lambda) was closer to one for all studied traits when two PCs were included as covariates, confirming that two PCs were the optimal number.

The significance Level (α) was adjusted using the Bonferroni correction, dividing the original significance level of 0.05 by the total number of tested SNPs (0.05/18417 = 0.0000027149) and was converted into -log_10_ (p) of 5.565 to improve data visualization.

The pairwise LD of SNP markers were calculated separately for each chromosome applying the full-matrix option in “TASSEL 5” software package (Bradbury et al., 2007). The results from TASSEL were used to estimate the LD decay over physical distance (Mbp) in R. The nonlinear model of Marroni et al. (2011) was applied to summarize the relationships between LD decay and physical distance (Marroni et al., 2011). Subsequently, a half-decay distance based on the maximum LD *R^2^* value and decay distance using a fixed LD threshold of 0.2 were found. QTL determination: MTAs were grouped as one part of the same QTL region when the LD (*R^2^*) coefficient was greater than 0.5.

GWAS was conducted using the Best Linear Unbiased Estimates (BLUEs) from individual trials and BLUEs across different years, as well as among different types of trials, to assess the stability and genetic pattern of resistance of found significant marker–trait associations (MTA).

To describe the effects of SNPs, we used three characteristics/metrics: allelic effects (β), PVE, and the coefficient of determination *R^2^* (adjusted).

Allelic effects (β) were computed using the BLINK model, and PVE was calculated using the following formula:


$$\frac{\beta^{2}*2*MAF(1-MAF)}{\sigma^{2}}$$

where:

MAF: Minor Allele Frequency of the SNP.

β: Estimated effect size of SNP.

σ²: Total phenotypic variance of the trait.

The coefficient of determination *R^2^* (adjusted) was estimated by fitting the Ordinary Least Squares (OLS) regression model using the *lm()* function in R. Furthermore, *R²* (adjusted) values were extracted and converted to percentages by multiplying by 100. The adjusted coefficient of determination is denoted as *R^2^* throughout the text.

Population analysis and identification of genotype clusters were performed using Principal Component Analysis (PCA) and hierarchical clustering based on 18,417 polymorphic SNPs. PCA was performed using the GAPIT package and BLINK method with default settings in R. The centering of genotypes was done without scaling using the *prcomp* function. Missing data was imputed with the middle values (Huang et al., 2019). The PCA plot was drawn using *ggplot2* package. Hierarchical clustering was based on the first two principal components of PCA. To perform hierarchical clustering, a distance matrix was computed using the Euclidean distance metric. Hierarchical clustering was performed using the *hclust* function with Ward’s method, and the *cutree* function was used to assign genotypes to clusters. Linkage distances were normalized to a percentage scale. The *factoextra* package was used to draw a dendrogram plot (Kassambara and Mundt, 2020).

Best linear unbiased estimates (BLUEs) were calculated for individual trials and across different combinations of trials using the META-R software (Alvarado et al., 2020). In the linear mixed model, genotypes were fitted as fixed effects, while environments, replicates, and genotype-by-environment interactions were treated as random effects. Broad-sense heritability (H²) was estimated using the following formula:


$$H^{2}=\frac{var(genotypic)}{var(phenotypic)}$$

The details of calculating broad-sense heritability (H²) mentioned in the article (Syed et al., 2024).

Calculating the *R^2^* (phenotype explained variation), the average means of the markers were used to replace NA, since replacing them with a major allele could slightly distort the effect of alleles.

To compare the genotypic responses between different clusters, violin plots with boxplots, jitter, and means were created using the *ggplot2* package in R. Pairwise Wilcoxon tests were performed to compare the genotypes responses to FHB between clusters. The *p*-values were adjusted using the Bonferroni method (Hollander et al., 2013).

Multiple regression analysis was performed using the *lm()* function from the base R package to determine the effects of MTAs/QTL on wheat phenotypic resistance (BLUEs across eight trials). Furthermore, the proportional contributions of major and minor alleles to the explained phenotypic variation were calculated using the LMG (Lindeman–Merenda–Gold) method via the function *calc.relimp()* from the package *relaimpo* in R (Grömping, 2007).

The visual displaying of major QTL on wheat chromosomes was built using an online tool “MG_2_C” for drawing genetic maps (Chao et al., 2021).

### 2.5 Candidate genes analysis

To identify putative candidate genes associated with FHB resistance, a list of genes in the genomic region within the identified QTL was extracted from the published spring wheat “Chinese Spring” RefSeq Annotation v1.1 (International Wheat Genome Sequencing Consortium (IWGSC), 2018) using a custom R script. The GO terms for the identified genes were obtained using the DAVID online tool (Sherman et al., 2022). GO term definitions were downloaded from https://geneontology.org/docs/download-ontology/ (The Gene Ontology Consortium et al., 2023). Combined protein families, domains, and the functional annotations (InterPro descriptions) were collected from EnsemblPlants (https://plants.ensembl.org/) connected to “Plants Genes 60” and “*Triticum aestivum* Refseqv2 genes” databases.

## 3 Results

Disease evaluation was performed using three types of inoculation: spray inoculation in 2022 and 2023 under field conditions and controlled conditions in 2023, spawn inoculation in 2022 and 2023, and point inoculation under controlled conditions. In individual trials, the heritability of FHB resistance was 0.68 on average, the highest heritability of 0.95 and 0.96 was found in the field spray trials in 2022 and 2023, respectively. The lowest heritability of 0.23 was observed in the spray inoculation trial under controlled conditions, and in the spawn inoculation trial in 2023 (0.28) (**Table 1**). Additionally, the heritability level increased when the BLUE values were calculated across several combinations of trials (**Table 2**). The phenotypic results of these experiments were reported in detail previously (Syed et al., 2024).

**Table 1:** The number of significant MTAs associated with resistance in individual trials.

MTA number	H^2^	Type of resistance	Abbreviation	Description
8	0.79	overall	FHBindex_2023	FHB index (2023)
6	0.95	overall	Field_spray_2022	Field spray (2022)
6	0.96	overall	Field_spray_2023	Field spray (2023)
1	0.77	II	Greenhouse_precise_2022	Greenhouse point (2022)
3	0.23	overall	Greenhouse_spray_2023	Greenhouse spray (2023)
3	0.76	I	Incidence_field_2023	Incidence field (2023)
2	0.68	overall	Spawn_severity_2022	Spawn severity (2022)
5	0.28	overall	Spawn_severity_2023	Spawn severity (2023)

*MTA = marker-trait associations, H^2^= broad-sense heritability (H^2^ values were obtained from Syed et al., 2024).

**Table 2:** The number of significant MTAs associated with FHB resistance across different combinations of the trials.

MTA number	H^2^	Type of resistance	Number of trials	Abbreviation	Description
11	0.82	Overall, I, II	8	8_env_all	2 spawn, 2 field spray, spray greenhouse, point inoculation greenhouse, FHB index, incidence
7	0.75	Overall, I, II	7	7_env_5plus_a	2 spawn, 2 field spray, spray greenhouse, point inoculation, incidence
12	0.85	Overall, I	7	7_env_b	2 spawn, 2 field spray, spray greenhouse, FHB index, incidence
10	0.82	Overall	6	6_env_c	2 spawn, 2 field spray, spray greenhouse, FHB index
10	0.78	Overall, I	6	6_env_b	2 spawn, 2 field spray, spray greenhouse, incidence
12	0.70	Overall, II	6	6_env_a	2 spawn, 2 field spray, spray greenhouse, point inoculation
12	0.72	Overall	5	5_env	2 spawn + 2 field spray + spray greenhouse
11	0.71	Overall	4	4_env	2 spawn+2 field
10	0.67	Overall	3	3_env	2 field spray + spray greenhouse
9	0.64	Overall	2	2_env_a	2 field spray
2	0.43	Overall	2	2_env_b	2 spawn

### 3.1 The population structure

Principal Component Analysis (PCA) was applied to investigate the population structure and detect the genetic patterns that explained the variation based on allele variation. The first principal component explained the largest amount of variation (9.2%). The first two principal components explained 15.1% of the variation, and the first three principal components explained 20.2%. The Elbow method demonstrated that two PCs capture the major genetic variation in our population and can be applied as covariates to adjust for population structural alleles (**Supplementary Figure S1**). Apart from that, the genomic inflation factor (λ) was closer to one when two principal components (PCs) were included as covariates, confirming that using two PCs as covariates resulted in sufficient correction for the population structure. Adding a third PC might not improve the findings, but could lead to overcorrection, potentially removing true associations.

The set of studied genotypes consisted of 10 exotic genotypes and 322 genotypes of European origin. To determine the number of clusters within the population, all genotypes were distributed according to their positions on the first two principal components, and the Elbow method was applied. According to the Within-Cluster Sum of Squares in the Elbow method, there were at least three subgroups within our population (**Supplementary Figure S2**). However, a cut-off point was selected at a 10% level of dissimilarity, assigning the genotypes into 13 clusters/subgroups for a detailed comparison of genetic relatedness between exotic resistant genotypes and adapted resistant genotypes (**Supplementary Figure S3**).

**Figure 1:**
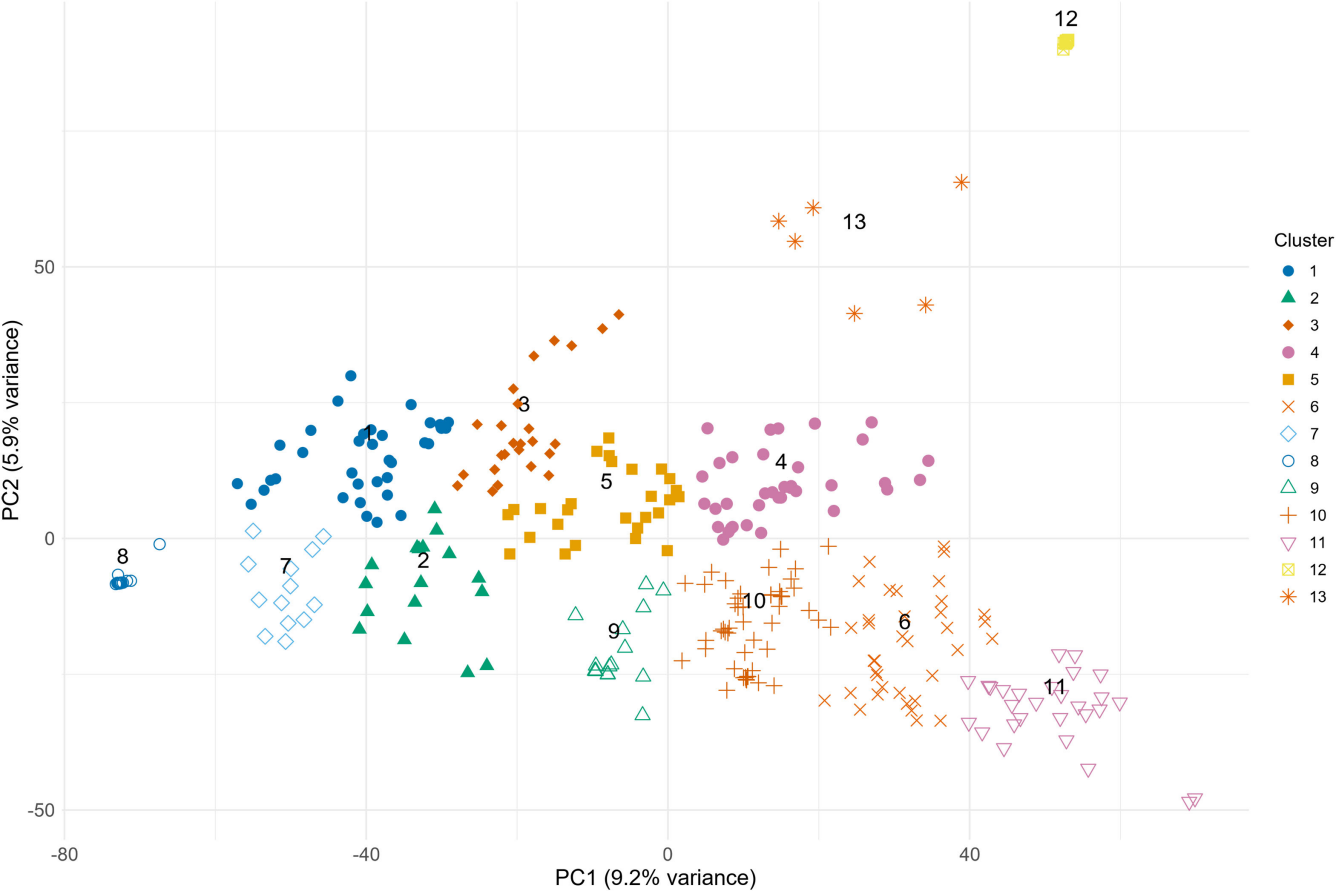
The PCA plot was developed based on 18,417 polymorphic SNPs, demonstrating the population structure of 332 spring wheat genotypes.

**Figure 2:**
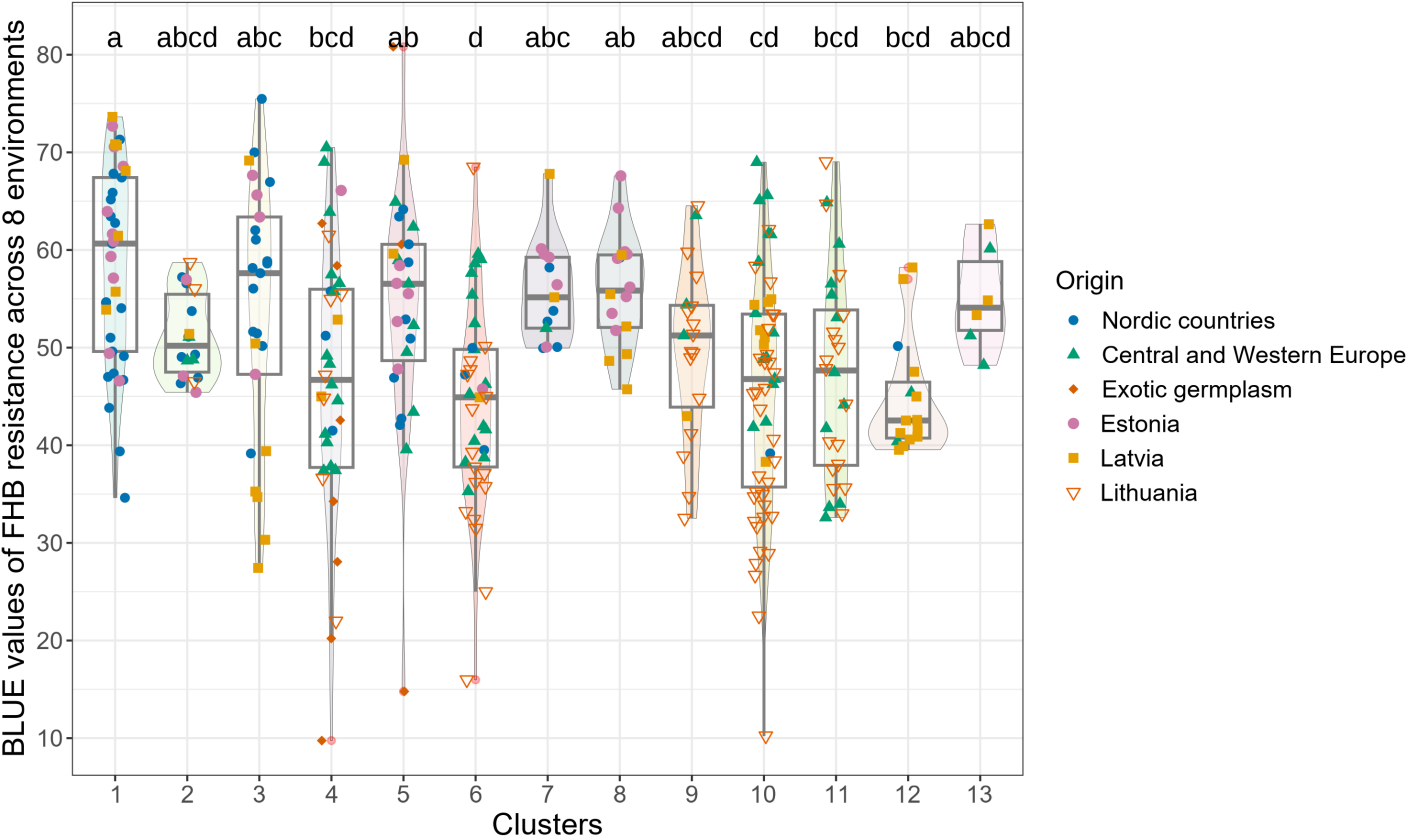
Comparison of clusters by phenotypic (BLUE) values for combined resistance (across eight trials).

Analysis of the genetic structure of the population based on 18,417 SNP markers demonstrated that Cluster 12 was the most distinct. Some distinctness was found for Clusters 13, 8, and 11 and, to a lesser extent, for Cluster 3. The other 8 clusters were relatively closely centered, indicating their close genetic relatedness (**Figure 1**). Among the 13 clusters, 2, 7, 8, 12, and 13 did not comprise any resistant or moderately resistant genotypes, while resistant fell closer to the middle of the population (**Figures 1**, **2**). Interestingly, exotic genotypes were intermixed with European genotypes and fell into two Clusters, 4 and 5, which were located in the middle of the population (**Figure 1**; **Supplementary Table S1**).

The most resistant genotypes were found within 4 Clusters: 3, 4, 5, 6, and 10. There was no clear relationship between the origin of genotypes, their resistance and genetic distinctness (based on 18,417 polymorphic SNPs). Resistant genotypes and genotypes of exotic origin did not form separate clusters (**Figure 2**). The clusters that comprised the most resistant genotypes also contained susceptible genotypes. Lithuanian genotypes prevailed among the most resistant, and few of them were of exotic and Latvian origin (**Figure 2**). Lithuanian breeding line DS-1577-8-DH does not have exotic relatives in the pedigree, had comparable levels of resistance to Sumai 3 (Syed et al., 2024) and belonged to the large genetic Cluster 10, which comprised both highly resistant and susceptible genotypes. Exotic resistant genotypes such as Wangshuibai, Gen020 (SHA3/CBRD), Gen066 (N894037), and the Lithuanian resistant breeding line DS-1401-6-DH and moderately resistant DS-570-2-DH and DS-1401-6-DH belonged to the same Cluster 4 (**Supplementary Table S1**). Clusters 3, 6 and 10 did not contain any exotic germplasms but comprised several resistant or highly resistant local genotypes. For example, Cluster 6 contained several resistant genotypes of Latvian origin. Clusters 6 and 10, comprised several resistant genotypes of Lithuanian origin. Genotypes which belonged to Cluster 1 had the highest susceptibility on average. According to the BLUEs values across the eight trials, the resistance of the genotypes in Cluster 1 differed significantly from that in Clusters 4, 6, 10, 11, and 12. Cluster 12 comprised mostly genotypes of Latvian origin with similar level of moderate resistance (**Figure 2**).

The LD pattern of the 332 spring wheat genotypes is depicted in a scatter plot of pairwise LD (*r^2^*) over physical distance in Mbp (**Supplementary Figure S4**). The average half-decay distance was 0.57 Mbp (when *r^2^* decreased to half of the maximum value) with the longest distance in the B genome (0.76 Mbp) followed by the D and A genomes (0.57 and 0.39 Mbp). The fastest LD decline was observed in chromosomes 2A and 6A (0.24 Mbp) and the longest LD was observed in chromosome 3B (1.03 Mbp) (**Supplementary Table S2**). The half-decay distances indicate that approximately half a million base pairs may belong to one LD block. Thus, on average, one SNP marker may be associated with about half a million base pairs. The level of genetic recombination was moderate in our population and consistent with other studies conducted on diverse wheat populations (Ghimire et al., 2022).

### 3.2 Association analysis between SNPs markers and FHB resistance

Significant MTAs were identified across all individual trials and among different combinations of trials. In total, 58 MTAs with known positions and seven with unknown positions were identified (**Supplementary Table S3**). The lowest number of MTAs was found in the greenhouse experiment, where point inoculation was used, and the largest number (12 MTAs) was found in the combinations of different trials (**Tables 2**, **3**).

**Table 3:** Significant QTL with *R^2^* larger than 10%.

QTL	SNP	Chr	Pos (bp)	-Log_10_ (*p*.value)	MAF	Allele Effect (%)	PVE, %	*R^2^*, %	Trials
*QFHB-2AL.1*	Tdurum_contig91519_224	2A	451809379	from 5.8 to 18.0	0.17	4.41 to 6.94	0.97-7.18	8.86-15.18	2_env_a, 3_env, 4_env, 5_env, 6_env_a, 6_env_b, 6_env_c, 7_env_a, 7_env_b, 8_env_all, Field_spray_2023
*QFHB-3AL.5*	AX-109493460	3A	743554170	6.3	0.36	5.61	2.58	10.35	Field_spray_2022
*QFHB-3AL.1*	Excalibur_c52772_1592	3A	341284496	from 6.8 to 9.9	0.13	2.87 to 4.14	1.61-2.95	13.37-15.07	8_env_all, 5_env, 7_env_b, 6_env_a
*QFHB-4AL.1*	AX-109477914	4A	590276039	7.3	0.08	5.64	0.50	11.92	2_env_field_spray
*QFHB-5AS*	tplb0056h22_2113	5A	48894696	9.1	0.19	3.61	2.49	11.02	7_env_b
*QFHB-2BL.2*	AX-94456169	2B	588688399	5.9	0.16	4.36	1.86	12.98	Incidence_field_2023
*QFHB-2BL.4*	BobWhite_c6365_965	2B	731894245	9.05	0.48	1.96	1.52	11.12	Spawn_severity_2023
*QFHB-2BL.1*	Excalibur_c29707_318	2B	412662314	from 5.8 to 12.9	0.21	from -2.79 to -4.57	0.73-3.28	10.49-13.94	2_env_a, 3_env, 4_env, 5_env, 6_env_a, 6_env_b, 6_env_c,7_env_b, 7_env_a, 8_env_all, Field_spray_2023
*QFHB-3BS*	AX-94428728	3B	299598299	from 6.16 to 6.19	0.11	6.53-6.56	0.85-1.26	12.43-12.52	FHBindex_2023, Field_spray_2023
*QFHB* *-6BL*	Tdurum_contig46828_730	6B	643165761	5.8	0.37	from -2.30 to -2.57	1.73-1.88	11.25-11.42	6_env_c, 5_env

* QTL = genomic region linked to variation in quantitative trait, SNP= Single nucleotide polymorphism used as SNP marker, Chr = Chromosome where specific QTL or SNP is located, Pos(bp) = the physical location of SNP in base pairs on chromosome, MAF = the frequency of minor allele, Allele effect = effect of major allele on the trait, *R^2^* = coefficient of determination.

*2_env_a (field spray of 2022 + 2023), 2_env_b (spawn grain of 2022 + 2023), 3_env (2-year field + greenhouse spray), 4_env (field spray 2022–23 + spawn grain 2022–23), 5_env (2-year field spray + 2-year spawn grain + greenhouse spray), 6_env_a (3 spray + 2 spawn grain + point inoculation), 6_env_b (3 spray + 2 spawn grain + FHB incidence), 6_env_c (3 spray +2 spawn grain + FHB index), 7_env_a (3 spray + 2 spawn grain + point inoculation + incidence), 7_env_b (3 spray + 2 spawn grain + incidence + FHB index), 8_env_all (3 spray + 2 spawn grain + point inoculation + incidence + FHB index).

Among 65 SNP markers, 12 explained more than 10% of the phenotypic variation according to the adjusted coefficient of determination *R^2^*). Among them, 7 MTAs had *R^2^* >10% and were stable (detected in more than 1 environment) (**Supplementary Table S3**).

Among 65 SNP markers, 12 explained more than 10% of the phenotypic variation according to the adjusted coefficient of determination (*R^2^*). Among them, 7 MTAs had *R^2^* >10% and were stable (were detected in more than 1 trial) (**Supplementary Table S3**).

**Figure 3:**
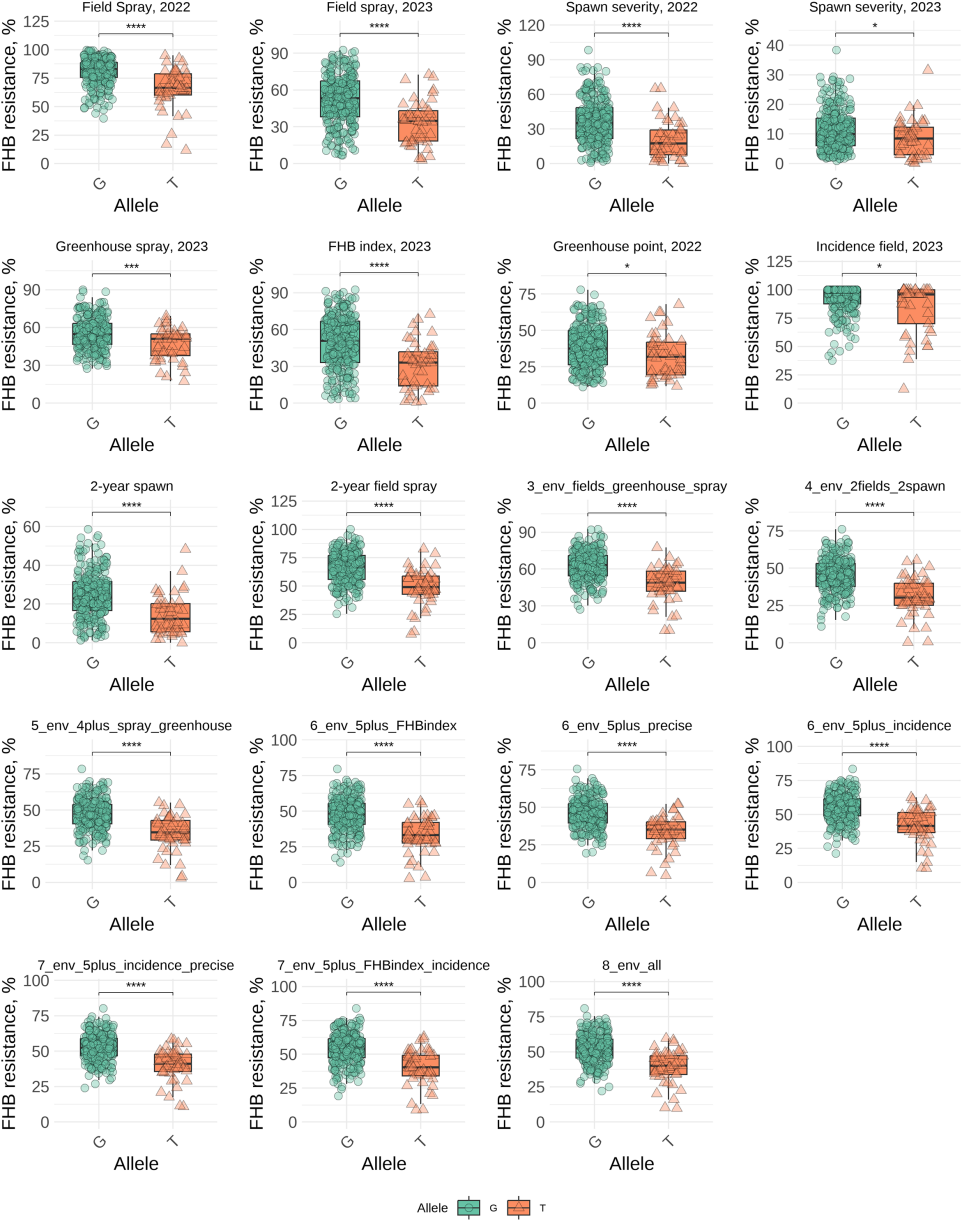
Comparison of FHB resistance (BLUEs) between genotypes carrying G and T alleles of the SNP marker (*Tdurum_contig91519_224*) in individual trials and across different combinations of trials. * - 0.01 < p ≤ 0.05, *** - 0.0001 < p ≤ 0.001, **** - p ≤ 0.0001.

MTAs were found across 15 chromosomes (1B, 2A, 2 B, 2D, 3A, 3B, 4A, 4D, 5A, 5B, 5D, 6B, 6D, 7A, and 7D) in the three wheat genomes. Among them, 25 MTAs were found in the A genome, 27 in the B genome, and six in the D genome. Cumulatively, across all the trials, not within a single trial, they explained 338%, 321%, and 37% of the variation (*R^2^*), respectively. The A and B genomes harbored the largest number of MTAs and explained a major part of the variation. Individual SNP analysis was performed for all significantly associated SNPs across the trials, where the associations were significant at the adjusted significance level of 5.565 (-log_10_). Comparing the BLUE values of groups with different alleles, the Wilcoxon test indicated that there were significant differences for 56 SNPs and no significant differences for nine SNPs. The results are presented in **Supplementary Table S3**. The strongest and most stable associations were found between the SNP marker Tdurum_contig91519_224 and FHB resistance. The allele effect of this marker ranged from 4.41% to 6.94%, PVE from 0.97% to 7.18%, and *R^2^* from 8.86% to 15.18%. The resistance to FHB was associated with minor allele “T” which had frequency of 17% in our panel, while most genotypes had “G” allele associated with susceptibility (83%). The difference in disease severity between the two groups ranged from 12.2% to 18.7% (**Supplementary Table S3**). The significant effects of this SNP marker were identified in nine different combinations of trials, demonstrating stable effects. However, a significant effect of this marker was identified in only one individual trial, namely field spray trial in 2023. A visual comparison of the groups with different alleles across eight individual trials and nine trial combinations is shown in **Figure 3**. Comparing the average values between groups with “G” and “T” alleles the Wilcoxon test demonstrated a significant effect of alleles in all trials and combinations, even in the trials where significant associations were not detected by GWAS (under a threshold of -log_10_(p) = 5.565). Significance at the level of *p* ≤ 0.05 between two groups was found in the following trials: spawn severity (2023), greenhouse point inoculation (2022), and incidence (2023); in other trials, the differences were significant at *p* ≤ 0.001 (**Figure 3**).

### 3.3 Accumulation effect of R alleles

**Figure 4:**
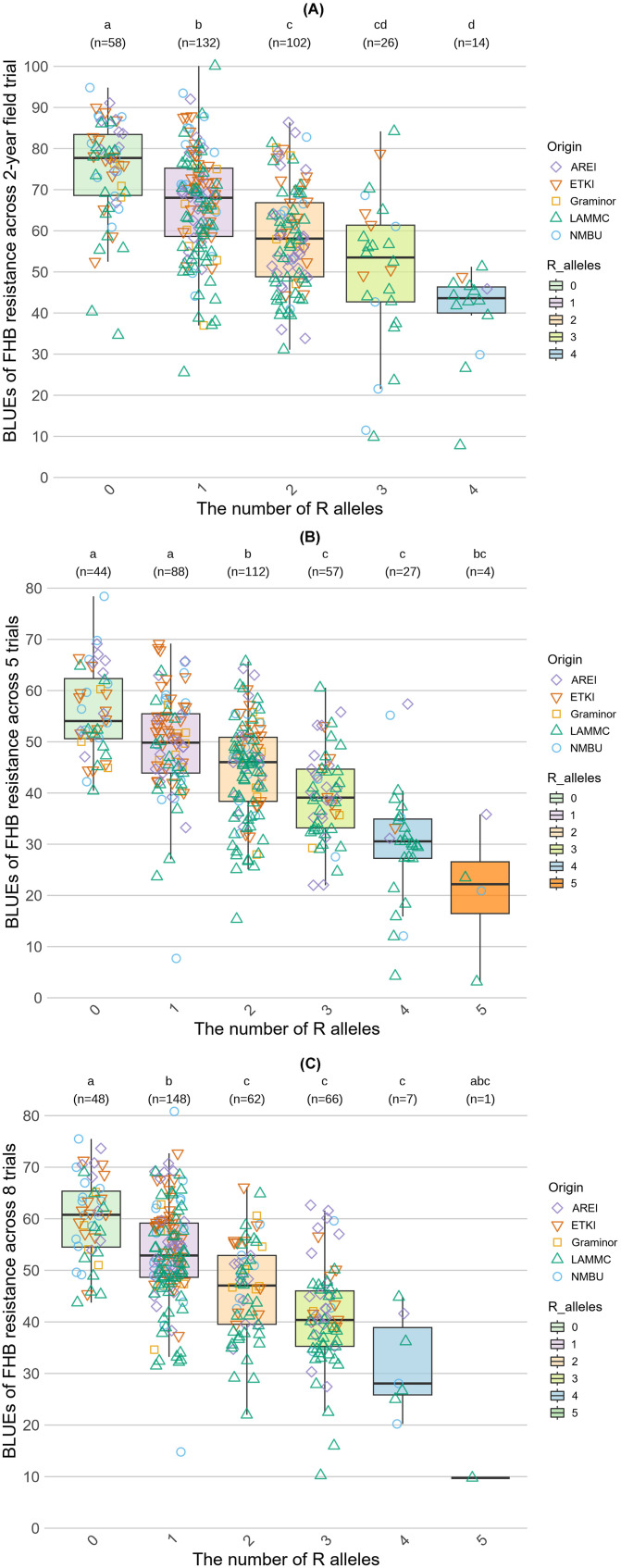
Accumulative effects of alleles associated with FHB resistance (*R* ^2^ > 5%). **(A)** represents the accumulation effect of R alleles across a 2-year spray field trial. **(B)** demonstrates the accumulation effect across five trials. **(C)** shows the accumulation effect across all trials. The letters above the plots indicate statistically significant differences between the groups according to the Kruskal-Wallis test (*p* < 0.05), followed by Dunn’s test for multiple *post hoc* pairwise comparisons with Bonferroni correction.

Up to 12 alleles significantly associated with resistance were found in the combinations when BLUE values were calculated across five, six, and seven trials and from one to eight alleles in the individual trials (**Tables 2**, **3**). However, many of these alleles provided minor effects (**Supplementary Table S3**). To select the most meaningful ones and analyze their cumulative effect an *R^2^* threshold of 5% was applied. Furthermore, to investigate the pyramiding effect of resistant (R) alleles, the wheat genotypes were divided according to the number of R alleles. The alleles associated with FHB resistance were labeled as “R alleles” and the number near the letter “R” indicates the number of associated alleles (**Figure 4**). Comparison of the average BLUE values of the groups according to the number of R alleles demonstrated a relationship between the number of R alleles and the level of resistance (**Figure 4**). The Kruskal-Wallis test (*p* < 0.05), followed by Dunn’s test for multiple *post hoc* pairwise comparisons with Bonferroni correction, were conducted among the genotype groups to determine if the differences were significant between groups. Across a two-year field spray trial, four MTAs (four R alleles) with *R^2^* effects above 5% were identified, and the level of resistance gradually increased depending on the number of R alleles. The average disease severity mean of the null group was 75.0%, the group with one allele had disease severity of 66.5%, while two, three and four alleles reduced disease severity to 58.6%, 50.1%, and 40.0%, respectively. The group that contained four R alleles was significantly more resistant than the groups with 0, one and two R alleles. The groups with two and three R alleles significantly differed from the groups with 0 and one alleles, and the group with one R allele significantly differed from the group with no R alleles (**Figure 4A**).

Across five trials of overall resistance, four MTAs with *R^2^* effects greater than 5% were detected. According to the average means of each group, an increased number of MTAs per genotype resulted in an improved resistance to FHB. The average severity level of the null group was 55.8%, while the severity levels for groups with increasing numbers of alleles were as follows: one allele (49.6%), two alleles (44.6%), three alleles (39.5%), four alleles (30%), five alleles (20.8%). The average means of groups 5R, 4R, and 3R significantly differed from the groups with 0, one, and two R alleles, and the groups 0R, 1R, and 2R significantly differed from each other (**Figure 4B**).

Across all trials, five significantly MTAs with *R^2^* effects greater than 5% were identified. Comparing the average means of each group, a clear relationship between the number of accumulated R alleles and disease resistance levels was observed. The average severity of the null group was 60.0%, while the severity levels for groups with increasing numbers of R alleles were as follows: one allele (53.2%), two alleles (46.5%), three alleles (41.0%), four alleles (31.8%), and five alleles (9.8%). The groups with two, three, four and five alleles differed significantly only from the groups 0 and 1R, while the average means of the groups 0 and 1R significantly differed from each other and the groups with two, three, four, and five R alleles (**Figure 4C**). The group that accumulated 5 alleles likely did not differ significantly from the other groups, because it was represented by only one genotype.

**Figure 5:**
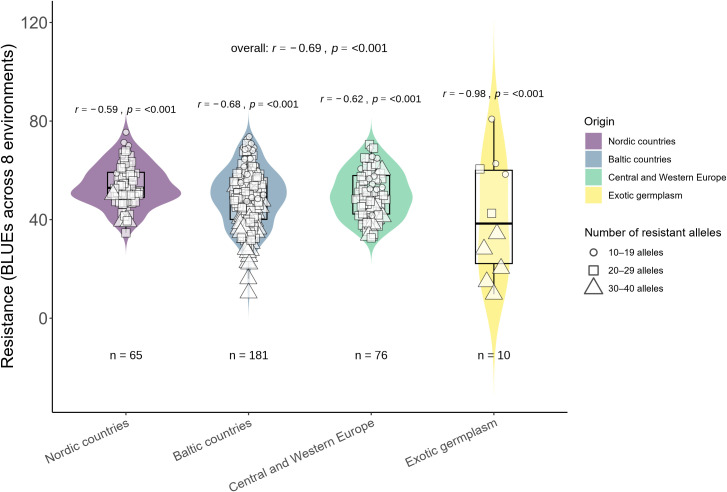
Relationship between resistance and the number of alleles (58 SNPs in total).

**Figure 6:**
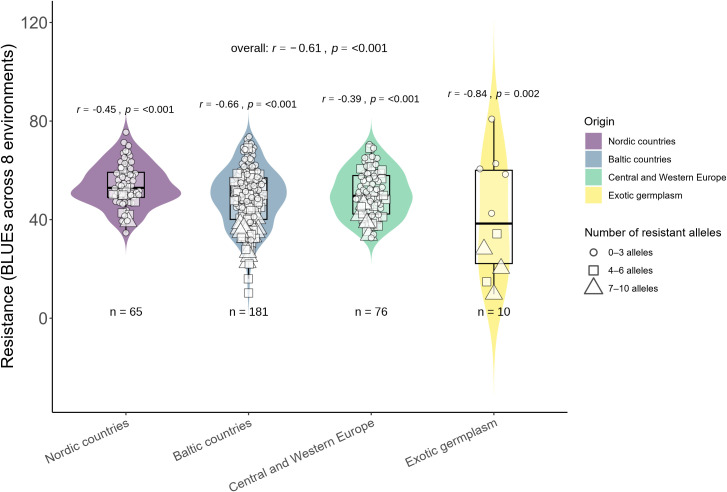
Relationships between resistance and the number of major-effect alleles (*R* ^2^>10%).

In total, 58 significantly associated MTAs were found across different individual trials and combinations of trials. Furthermore, the relationships between the number of these MTAs and BLUE values across eight trials were determined. Additionally, the genotypes were grouped according to the number of MTAs and their geographic origin (**Figure 5**). Multiple regression analysis was performed to determine the effect of 58 MTAs and partial contributions of 10 major and 48 minor MTAs. Collectively, 58 MTAs explained 55% of the variation in resistance. A larger part of this variation was explained by 10 major MTAs (31%), while 48 minor MTAs were responsible for the remaining 24% (**Supplementary Figure S5**). Most of these MTAs were unstable, identified in single trials (or across a specific combination of trials), and demonstrated only minor effects (**Supplementary Table S3**). Nevertheless, a relatively strong correlation (*r*=0.69, *p* < 0.001) and linear regression coefficient of determination (adjusted *R^2^* = 0.55) were found between the number of resistant alleles and BLUEs across eight trials (**Figure 5**, **Supplementary Figure S5**). The most resistant cultivars derived from Baltic countries, and a few genotypes had an exotic origin (**Figure 5**). Additionally, the associations were determined only for major MTAs (using a threshold of *R^2^* >10%) to determine the overall effect of major MTAs (**Figure 6**). Ten major-effect MTAs explained the largest part of resistance (*r*=0.61, *p* < 0.001; *R^2^ =* 0.31) (**Figure 6**, **Supplementary Figure S5**). However, considering only major QTL, some genotypes fell into the tails of violins as most resistant, but belonged to the second group possessing only 4–6 MTAs (**Figure 6**). In contrast, when all MTAs were considered, the most resistant genotypes (in the tails of violins) belonged to the Group 3 with the highest number of MTAs (**Figure 5**). Overall, 48 minor MTAs explained 24% of the variation, indicating that small-effect MTAs also contributed significantly to resistance.

### 3.4 QTL determination

Across all trials and their combinations, we identified a total of 65 MTAs, 58 of which had known physical positions within the genome (**Supplementary Table S3**). To define QTL regions associated with FHB resistance, half-decay chromosome-specific distances were applied (**Supplementary Table S2**). If the LD values were higher than 0.5 between nearby MTAs, they were assigned to one QTL. Overall, 55 QTL were defined (**Supplementary Table S4**), 10 of which had *R^2^* coefficients higher than 10% (**Table 3**). Among them, 51 were related to overall resistance, and only one *QFHB-2D* was associated with Type II resistance (*R^2^* value was 6.2%). Three QTL were associated with Type I resistance *FHB-2B.3*, with an *R^2^* value of 13%, and *QFHB-7A* with an *R^2^* value of 6%, while the third *QFHB-2BS* was related to both Type I resistance and overall resistance (**Supplementary Table S4**).

To assess the correspondence between the identified QTL and previously published ones, we compared their physical positions. For comparison, we used a set of published QTL from 2000 to 2020 compiled by Zheng et al. (2021). The authors collected 625 FHB associated QTL from 113 publications and determined their physical positions by aligning flanking and peak markers to the Chinese Spring reference genome, RefSeq v1.1 (International Wheat Genome Sequencing Consortium (IWGSC), 2018) (Zheng et al., 2021).

Comparing the physical positions of QTL identified in this study with previously published, we found that 46 QTL were collocated with previously reported QTL (**Supplementary Table S5**), whereas 8 QTL did not match any published QTL, 4 of them (AX-89483131, TA001900-1836, AX-158584923 and IAAV6297) had *R^2^* values above 5% and the difference between the two groups of alleles was significant at *p* ≤ 0.001. AX-95100505 and AX-158585120 had *R^2^* values of 2.87% and 2.65%, respectively. AX-111556997 and TA016804–1075 had *R^2^* values close to 0% and there was no significant difference between the two groups of alleles. Meanwhile, all 10 QTL with phenotypic variation (*R^2^*) higher than 10% collocated with previously reported QTL (**Table 4**).

**Table 4:** Collocation of QTL (*R^2^* > 10%) identified in this study with previously published QTL based on physical positions.

QTL in this study		Previously published*		
Name	*R^2^* effect	Reference	Population	PVE or *R^2^*
*QFHB-4AL.1*	11.92	Szabó-Hevér et al., 2012	DH (Frontana/Remus)	9
		Petersen et al., 2016	F5RIL (NC-Neuse/AGS 2000)	11.5
*QFHB-3AL.5*	10.35	Gervais et al., 2003	F7RIL (Renan (resistant)/Recital (susceptible)	6.2
*QFHB-3BS*	12.43-12.52	Zhang et al., 2004	F7RIL (Wangshuibai/Alondra)	15.7
		Zhang and Mergoum, 2007	F5RIL (Sumai 3/Stoa)	12.4
		Holzapfel et al., 2008	RIL (Apache/Biscay)	2.1
		Zhang et al., 2012a	F6RIL (Baishanyuehuang/Jagger)	8.1
		Islam et al., 2016	F9RIL (Truman/MO 94-317)	10.3
		Shah et al., 2017	F2 (Zhoumai-27/Shengxuani-6)	7.29
*QFHB-2BL.2*	12.98	Xue et al., 2010	RIL (Nanda2419/Wangshuibai)	17.9
		Zhang et al., 2018	DH (FL62R1/Stettler)	16.2
*QFHB-2BL.4*	11.12	Eckard et al., 2015	RIL (Wheaton (PI 469271)/Sapporo Haru Komungi Jugo (PI 81791)	3.91
*QFHB-3AL.1*	13.37-15.07	Cai and Bai, 2014	F5RIL (HCD/Jagger)	7.5
		Zhang et al., 2012a	RIL (Heyne/Trego)	14
		Shen et al., 2003	F5RIL (Patterson/F201R)	13
		Zhang et al., 2012b	F6RIL (Baishanyuehuang/Jagger)	4.8
		Buerstmayr et al., 2013	F6BC1 (Mt. Gerizim #36/Helidur)	22
*QFHB-2AL.1*	8.86-15.18	Zhang et al., 2014	F8RIL (Ben/PI41025)	8
*QFHB-5AS*	11.02	Chen et al., 2006	DH (W14/Pion2684)	16
		Li et al., 2012	F8RIL (HFZ/Wheaton)	6.9
		Ágnes et al., 2014	DH (GK Mini Mano/Frontana)	12.2
		Eckard et al., 2015	F2: Wesley-Fhb1-BC56	7.5
		Ren et al., 2019	F6RIL (Luke/AQ)	9.8
		Chen et al., 2006	DH (W14/Pion2684)	8
		Jiang et al., 2007	F7RIL (Veery/CJ 9306)	5.2
		Lu et al., 2013	F6RIL (SHA3/CBRD/Naxos)	10.8
*QFHB-2BL.1*	10.49-13.94	Gervais et al., 2003	F7RIL (Renan (resistant)/Recital (susceptible)	12
		Somers et al., 2006	DH (strongfield/blackbird)	26
		Xue et al., 2010	RIL (Nanda2419/Wangshuibai)	17.9
		Islam et al., 2016	F9RIL (Truman/MO 94-317)	16.1
*QFHB-6BL*	11.25-11.42	Lin et al., 2004	F6RIL (Nanda2419/Wangshuibai)	17.8
		Szabó-Hevér et al., 2012	DH (Frontana/Remus)	6.8
		Buerstmayr et al., 2013	F6BC1 (Mt. Gerizim #36/Helidur)	22
		Ágnes et al., 2014	DH (GK Mini Mano/Frontana)	20.5
		Buerstmayr and Buerstmayr, 2015	F5RIL (Capo/Arina)	5.4

*the list of published QTL (from 2000 to 2020) and their physical positions were determined by Zheng et al., 2021 by aligning flanking and peak markers to the Chinese Spring reference genome, RefSeq v1.1 (IWGSC, 2018).

Additionally, we reviewed recent GWAS that reported associations with wheat resistance to FHB. Overall, 1760 MTAs were found in 13 articles (**Supplementary Table S5**). To evaluate the potential collocation of our QTL with previously published MTAs, we manually added ±20 Mb to the positions of the markers. It was found that 42 QTL were potentially related to the MTAs from previous GWAS studies. When both published QTL and MTAs were compared with the QTL from this study, only 2 QTL (*QFHB-4DS* and *QFHB-7DS.1*) were not collocated in physical positions and are presumably novel (**Supplementary Table S7**). *QFHB-4DS* was found only in one individual trial (spawn 2022) with an *R^2^* value of 7.68%, and the difference between the two groups of different alleles was 11.67% at the 0.001 significance level, while *QFHB-7DS.1* was found in one individual trial (FHBindex_2023) with an *R^2^* value close to 0%, and no significant difference between the two groups was found.

### 3.5 Candidate gene identification

Using annotated wheat genome data for high-confidence genes from the International Wheat Genome Sequencing Consortium (IWGSC) RefSeq v1.1 (International Wheat Genome Sequencing Consortium (IWGSC), 2018), 705 gene names and their physical positions were extracted from the physical regions of 55 identified QTL. Furthermore, 12590 matching GO terms were found for 543 genes (**Supplementary Table S8**). Alongside, significantly up- or downregulated genes from previous transcriptomic studies were collected, in which gene expression was analyzed by comparing resistant and susceptible genotypes/groups in response to inoculation with *Fusarium graminearum*, to assess their correspondence with identified genes in this study (Buerstmayr et al., 2021; Pan et al., 2018; Seifi et al., 2023). Subsequently, 52 genes could be referred as candidate genes, since they matched both our identified genes and those reported in transcriptomic studies as significantly associated with FHB. These 52 genes were found within 25 QTL identified in our study (**Supplementary Table S9**). Most genes were downregulated, 36 exhibited significantly decreased expression, while only 16 were upregulated, which is consistent with findings from previous studies (Buerstmayr et al., 2021; Seifi et al., 2023). Notably, several genes were found within the same QTL. For example, eight candidate genes were found within *QFHB-1BS.2*, seven within *QFHB-1BS.1*, six within *QFHB-1BS.3*, four within *QFHB-3BL.4*, three within *QFHB-4DS* and *QFHB-2BL.1*, and two within *QFHB-2AL.5*, *QFHB-2DL*, and *QFHB-3AL.5* (**Supplementary Table S9**).

Some candidate genes were inside or within close proximity to SNP markers, such as TraesCS1B02G324300, TraesCS1B02G046300, TraesCS3A02G527200, and TraesCS1B02G075200, which were merely 122, 1129, 1452, 1787 bp away from the SNP markers (**Supplementary Table S9**). The identified candidate genes encoded proteins that play key roles in pathogen detection and defense signaling. For example, TraesCS3B02G598200, TraesCS3B02G598400, TraesCS1B02G047000 and TraesCS1B02G324300 contribute to cell wall reinforcement. TraesCS5A02G367100, TraesCS2B02G536500, TraesCS1B02G046000, TraesCS1B02G046300, TraesCS1B02G114100, TraesCS6B02G052400, TraesCS3A02G530200 and TraesCS2B02G296300 participate in pathogen recognition that can be referred to as Type I resistance. TraesCS1B02G114300, TraesCS1B02G113600, TraesCS1B02G113800, and TraesCS5B02G426400 were associated with regulation of ROS and programmed cell death that hints to Type II resistance. Other genes were associated with Type III resistance, such as DON detoxification (TraesCS3B02G598200 TraesCS3B02G598400, TraesCS1B02G047000, TraesCS1B02G114300). Moreover, some genes (TraesCS5A02G367100, TraesCS1B02G114100, TraesCS3B02G596200) play putative roles in susceptibility pathways, such as calcium signaling or receptor-like kinases (RLKs). Functional description of all the identified genes are presented in **Supplementary Table S9**.

## 4 Discussion

### 4.1 Genome-wide association analysis

Fusarium head blight (FHB) is a challenging threat to wheat production worldwide. Resistance to FHB is regulated by a complex network of small-effect genes (Buerstmayr et al., 2021). In previous studies, plant breeders and researchers relied on genetic tools that enabled them to work with a limited number of genes or QTL. Traditional genetic methods were limited to screening large genomic regions, which is crucial for investigation of complex traits such as FHB resistance. However, after the development of high-density genotyping techniques such as high-density SNP arrays and genotyping-by-sequencing (GBS) combined with Linkage Disequilibrium analysis, research on FHB resistance has gained momentum, opening new possibilities to develop improved disease resistance in wheat (Mir et al., 2023; Myles et al., 2009; Saini et al., 2021). Using LD analysis, which can be performed directly in a diverse breeding population, a large part of the wheat genome can be quickly analyzed for associations to FHB resistance without the need for the whole-genome resequencing. Furthermore, physical regions that include the responsible genes can be defined by performing LD analysis. Although LD does not provide direct information about genetic linkage, it provides statistical evidence of linkage between alleles based on their actual distribution. That is not completely random, the frequency can be affected not only by genetic linkage but also by genotype pedigree, adaptability to specific environments, and breeding selection (Flint-Garcia et al., 2003; Sahoo et al., 2022; Scherer and Christensen, 2016). The use of LD strength between markers in diverse populations provides quick LD-based detection of QTL regions; however, the physical regions are not fine-mapped, do not have precise boundaries, and are based on selected statistical probability levels.

### 4.2 QTL mapping in diverse and bi-parental populations

Traditionally, QTL identification is performed using mapped populations such as recombinant inbred lines (RILs), near-isogenic lines (NILs), or doubled haploids (DHs) (Zheng et al., 2021). Alternatively, GWAS, which is based on high-density genotyping of diverse populations, is usually used to identify MTAs without explicitly defining the specific QTL regions. In GWAS, when genetically diverse populations are used, the structural alleles can increase the risk of detecting false associations (Mir et al., 2023). When mapping populations in which all progenies are genetically related are used for analysis, the detection of associations between markers and traits is more reliable. However, the development of mapping populations requires significant time and effort. Because of the lower frequency of recombination events in such populations, the linkage disequilibrium decay is slower, and defined QTL regions are relatively large. Conversely, the application of diverse populations makes it possible to use the existing breeding populations. The higher frequency of recombination events in diverse populations results in higher LD decay. In our study, LD-based detection of QTL region was used. The main purpose of defining approximate QTL regions based on chromosome-specific half-decay distance was to set the searching boundaries for candidate genes and to check the collocation between relatively small QTL identified in diverse populations and previously detected QTL from mapping populations.

### 4.3 Overview of identified MTAs and QTL

Overall, 58 MTAs with known positions and seven with unknown positions were identified in the studied wheat panel. A comparable number of identified MTAs was found in previous GWAS studies of FHB resistance in wheat, when diverse populations were studied (Ghimire et al., 2022). In contrast, mapped biparental populations do not possess such diversity of favorable MTAs/QTL, and the number of identified QTL is usually several times smaller in biparental populations than in diverse populations (Zheng et al., 2021). Applying half-decay chromosome-specific distances, 55 QTL were determined in the spring wheat population. Three pairs of MTAs were merged because they were strongly associated, LD values between them varied from 0.94 to 1.0. By comparing the physical collocation of the QTL identified in this study with previously detected QTL from mapped populations using chromosome-specific half-decay distances, we surprisingly found that 46 out of 55 were collocated with known QTL. This might indicate that the significantly associated markers were relatively closely positioned to the causal genes, and the LD approach of defining boundaries of QTL can be applied for approximate determination. Almost all identified QTL were associated with overall resistance. Only *QFHB-2D* (AX-94872625) was associated with Type II resistance after point inoculation under controlled conditions, with an *R^2^* value of 6.2%. Three QTL associated with Type I resistance: *QFHB-2B.3* (AX-94456169) with *R^2^* value of 13%, and *QFHB-7A* (AX-158591608) with *R^2^* value of 6%, which are presumably related to the initial regulation of wheat defense. The third *QFHB-2BS* (BobWhite_c8113_532) was connected not only with initial resistance but was associated with later resistance and found in ten different combinations of trials (**Supplementary Table S3**). The remaining 51 QTL were related to overall resistance. Crossing wheat cultivars that possess different types of resistance might be an effective strategy to combine the different components of resistance. For example, the breeding line Gen323 (DS-1401-6-DH) possesses three QTL of Type I resistance and nine QTL of overall resistance (across five trials of overall resistance) but does not contain QTL of Type II resistance. Crossing this breeding line with other breeding lines that already possess Type II resistance allele, along with a complex of overall alleles (e.g. breeding line Gen322), might result in the development of transgressive segregates with improved FHB resistance.

Some minor QTL from this study collocated with QTL with relatively high effects in other studies. For example, *QFHB-2DL* had *R^2^* value of 6.18 in our study, while in other studies, its effect ranged from 12.6 to 18 (**Supplementary Table S5**). It is noteworthy that the most stable and effective QTL, *QFHB-2AL.1*, collocated with only one published QTL by Zhang et al. (2014). However, the authors defined a large region from 341.9 Mbp to 534.9 Mbp indicating 193 Mbp length/window at 2A chromosome and used mapping population, while in our study we defined regions from 451.6 Mb to 452 Mbp using only 0.48 Mbp window. Moreover, no genes related to FHB resistance were found within *QFHB-2AL.1* and *QFHB-3AL.1*, according to the GO terms (**Supplementary Table S8**) and InterPro descriptions (**Supplementary Table S10**). Presumably, resistance genes were located outside these defined QTL. When the region of QTL is defined based on LD, the causal genes are more likely to be within this region; however, they can still be out of the defined region. Therefore, additional analysis of QTL regions is required.

### 4.4 Candidate genes and resistance mechanisms

In total, 705 genes were extracted from the physical regions of 55 identified QTL. Matching significantly up- and downregulated genes from the transcriptomic studies (Buerstmayr et al., 2021; Pan et al., 2018; Seifi et al., 2023) with genes located within determined QTL, 52 candidate genes were found. Among them, 36 (69.2%) were downregulated and 16 (30.8%) were upregulated in response to inoculation with FHB (**Supplementary Table S9**), which is consistent with the findings of previous studies (Buerstmayr et al., 2021; Seifi et al., 2023). We did not find any functional validation of these 52 candidate genes, such as gene silencing or overexpression, in published literature to prove the true relationship between these genes and FHB resistance. However, some identified candidate genes encode wheat proteins, the roles of which have been well studied in wheat defense against FHB, supporting their potential contribution to FHB resistance (Ma et al., 2022; Sirangelo, 2024). For instance, TraesCS3B02G598200 encodes a glycosyltransferase that plays a vital role in DON detoxification by modifying it into DON-3-glucoside, cell wall reinforcement, and hormone regulation (Ghimire et al., 2022; Gottwald et al., 2012; He et al., 2018; Lemmens et al., 2005). TraesCS1B02G114300 encodes Glutathione S-transferase (GST) that is also a well reported protein for FHB resistance, associated with defense activation, detoxification, and oxidative stress (ROS). Additionally, it is an antioxidant that contributes to minimizing PCD (Gullner et al., 2018) by forming DON-glutathione conjugates, and aids in DON detoxification (Edwards et al., 2000; Gardiner et al., 2010; Seifi et al., 2023). TraesCS5A02G367100 and TraesCS2B02G536500 are encoded for receptor-like protein kinases (RLKs). These proteins are paramount for plant growth, strengthening of the cell wall, oxidative stress responses, and activation of defense signaling. Protein kinases play a crucial role in signaling when a pathogen is identified (Romeis, 2001; Tang et al., 2017). Furthermore, TraesCS7A02G051800 is identified as a 2-oxoglutarate/Fe(II)-dependent oxygenase (2OG oxygenase) superfamily enzyme involved in secondary metabolite synthesis (Jia et al., 2017). Inspection of the functions of candidate genes might provide additional insights into their resistance mechanisms and facilitate the proper pairing of QTL to attain durable FHB resistance. In our analysis, it was found that some QTL contributed to basal defense, while others participated in the detoxification of FHB mycotoxins (**Supplementary Table S9**). Seifi et al. (2023) has previously postulated that if PCD is inhibited but DON production is not limited, the visual symptoms may be reduced, nevertheless, a high level of mycotoxins would be present. Conversely, if DON production is limited via the activation of ROS scavengers, and plant cell death process is not limited, visual symptoms would be visible; however, the levels of DON would be low in the grain. Therefore, breeders should aim to develop such resistance when both DON production and PCD are limited (Seifi et al., 2023).

### 4.5 Contribution of identified QTL and the pyramiding effect

The results of the current study confirm the commonly accepted statement that wheat resistance to FHB is controlled by multiple number of small-effect QTL, and the determination of which is hampered by environmental effects (Mesterhazy, 2024; Miedaner et al., 2024). In this study, the GWAS was conducted using phenotypic data obtained from different inoculation methods under field and controlled conditions. Moreover, GWAS was carried out not only within individual trials but also across different combinations of trials that extended the number of identified significantly associated QTL. Altogether, 55 QTL were identified, most of which had small effects and were found only in one individual trial or in one combination of trials (**Supplementary Table S3**). The association between the number of QTL and FHB resistance was relatively strong, considering that FHB resistance is highly complex, some QTL related only to one type of resistance (Type I, Type II or overall), and were determined only under specific inoculation methods or environments. A relatively strong Pearson correlation (*r*=0.69, *p* < 0.001) and linear regression coefficients of determination (adjusted *R^2^* = 0.55) were found between the number of resistant alleles and the BLUE values of resistance across eight trials (**Figure 5**). Consequently, the effect of 10 major QTL was 31% (*R^2^ =* 0.31), while the effect of 45 minor QTL was 24% (*R^2^ =* 0.24) on the phenotypic variation (**Supplementary Figure S5**). The results demonstrate that even not stable “hardly detected” minor QTL are essential for wheat resistance to FHB and should not be neglected. The range of accumulated favorable QTL varied from 10 to 40 per genotype, while the number of major QTL varied from 0 to 10. The results demonstrate that these major QTL explained a large part of the variation and could potentially facilitate wheat breeding for FHB resistance. Additionally, this might indicate that modern cultivars possess plenty of favorable small-effect QTL, and new superior genotypes can be developed due to the transgressive segregation using selection under high disease pressure. The development of improved FHB resistance through transgressive segregation was observed by Mesterházy et al. (2018) and Zhang et al. (2018). Furthermore, the findings indicate that the inheritance of resistance to FHB had an accumulative nature. The more favorable QTL stacked, the greater the resistance to FHB. The positive additive effect of pyramiding small-effect QTL for improving wheat resistance has been reported in many studies (Haile et al., 2023; Kirana et al., 2023). For example, in the study by Ghimire et al. (2022) investigating the resistance of 278 elite winter wheat breeding lines, 11 QTL with *R^2^* greater than 10% were detected using GWAS. The severity of FHB was reduced by 21.6% when breeding lines possessed three alleles associated with FHB, a combination of four alleles resulted in a reduction of 32.6%, and a combination of five alleles caused a reduction of 42.6% compared to the null group (Ghimire et al., 2022). However, it should be noted that in this study some genotypes possessed close to the maximum number of favorable alleles, whereas they had an average or even below-average level of resistance (**Figure 5**). These results might suggest that there were other factors influencing resistance, such as phenotyping inaccuracies caused by environmental effects, the presence of additional unidentified FHB-related QTL, epistatic gene interactions, or epigenetic regulations that contributed to the expression of FHB resistance (Brar et al., 2019; ElDoliefy et al., 2024; Kumar et al., 2020; Mierziak and Wojtasik, 2024).

### 4.6 Summary and conclusions

The largest limitations in wheat breeding for FHB resistance are the lack of adapted sources of resistance and the genetic linkage between resistance and undesirable agronomic traits. The widely utilized Chinese sources of resistance, such as Sumai 3 and Wangshuibai, possess inferior agronomic traits and do not perform well in the environments of the Baltic and Nordic countries. Therefore, the search of resistance for ‘native’ resistance within locally adapted germplasm is an alternative breeding strategy for FHB resistance (Brown-Guedira et al., 2008; Mesterházy et al., 2018; Thambugala et al., 2020). Phenotyping a panel of 332 spring wheat varieties and breeding materials demonstrated that FHB resistance can be found in adapted genotypes to these regions (Syed et al., 2024). The medium resistance was found in breeding materials from general breeding and the most resistant breeding lines were detected in a subset of resistant breeding lines (breeding lines from Gen303 to Gen336) (Syed et al., 2024) which was previously selected after one or two cycles of screening under artificial inoculation. Some of these lines had resistance comparable to that of Sumai 3 (Syed et al., 2024). In our study, GWAS revealed ten major favorable alleles in wheat genotypes adapted to the environments of Baltic and Nordic countries. Although most of the identified QTL were found within previously published genomic regions, it was demonstrated that resistance to FHB can be found in the European gene pool, and moderate to high levels of resistance are controlled by the additive effect of major and small-effect QTL. A GWAS conducted on a large collection of adapted genotypes to Baltic and Nordic countries enabled identification of QTL, which presumably do not possess a negative linkage with crucial agronomic traits because they were selected within adapted breeding material. The accumulation of QTL identified in this study and the utilization of breeding lines carrying resistance alleles will facilitate further genetic improvement of wheat resistance to FHB in the environments of the Baltic and Nordic countries. These findings demonstrate that FHB resistance in wheat cultivars for these regions can be further improved by pyramiding these R alleles.

In conclusion, 55 QTL for wheat resistance to FHB were identified in the European gene pool. Among 55 QTL associated with FHB resistance, 10 had major effects (*R^2^* >10%), two QTL (*QFHB-4DS* and *QFHB-7DS.1*) have not been previously published and are presumably novel. The majority of QTL (52 QTL) were associated with overall resistance, one QTL (*QFHB-2DL*) was linked with Type II resistance, and two QTL (*QFHB-7AS* and *QFHB-2BL.2*) were associated with Type I resistance. According to the principal component analysis performed on the basis of 18,417 SNPs, resistant genotypes did not fall into separate clusters, but were placed in Clusters 3 and 4 in the middle of the population. Cluster analysis demonstrated that exotic resistant genotypes did not form a separate cluster, resistance was fairy well distributed within the population, and it can be found within the European wheat gene pool. Pyramiding of three and more major QTL resulted in improved resistance. A total of 52 candidate genes were identified by analyzing genes near significantly associated SNPs in combination with published transcriptome data. All identified QTL were found in elite, adapted breeding materials, and can be utilized in wheat breeding for improving FHB resistance in the Baltic and Nordic regions.

## Data Availability

The original contributions presented in this study are included in the **Supplementary Material**. Further inquiries can be directed to the corresponding author.

## Supplementary material

Supplementary Figure 1 Determination of the optimal number of principal components (PCs) using the Elbow method.

Supplementary Figure 2 Estimation of the optimal number of clusters using the Elbow method.

Supplementary Figure 3 A dendrogram of 332 genotypes using hierarchical cluster analysis of 18,417 polymorphic SNPs.

Supplementary Figure 4 Scatter plot of genome-wide linkage disequilibrium (LD) (*r* ^2^) versus physical distance (Mbp) for the 332 spring wheat genotypes based on 18,417 SNP markers within whole genome. The fitted locally weighted polynomial regression-based (LOESS) curve is indicated. The red dashed lines demonstrate the intersection between the critical LD value *r*² = 0.2 and physical distance.

Supplementary Figure 5 Proportion of explained variation by three QTL groups: 55 QTL, 10 major and 45 minor in wheat resistance (BLUEs across eight trials)

Supplementary Figure 6 Distribution of major QTL on wheat chromosomes based on RefSeq v1.1.
