# Supplementary figures and images for: GWAS analysis of Fusarium head blight resistance in a Nordic-Baltic spring wheat panel
Source: Front Plant Sci. 2025 Jul 23;16:1604296. doi: 10.3389/fpls.2025.1604296 (PMC12325435; doi:10.3389/fpls.2025.1604296)

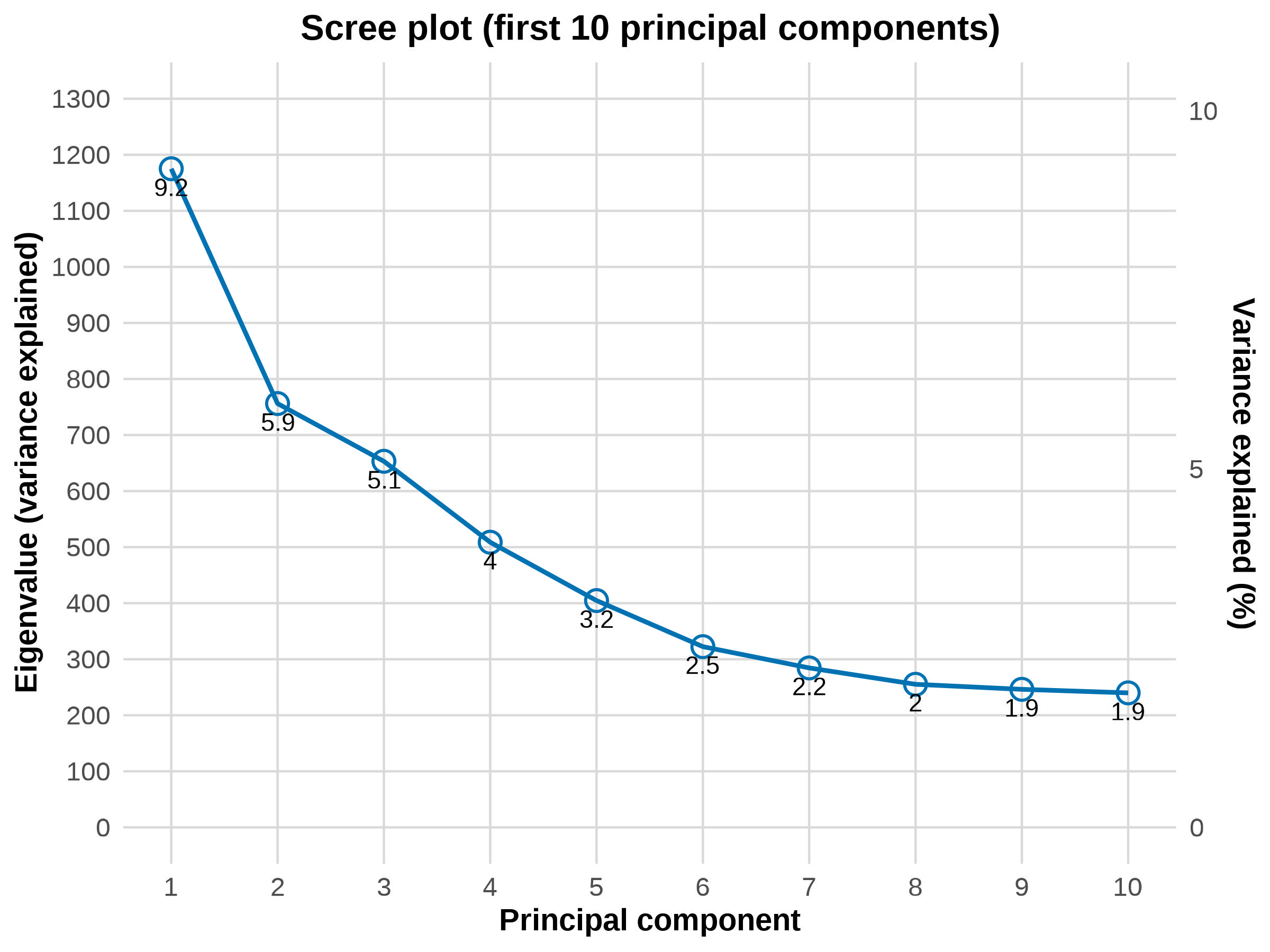

Supplement: Supplementary Figure 1 — Determination of the optimal number of principal components (PCs) using the Elbow method. [file Image1.jpg]

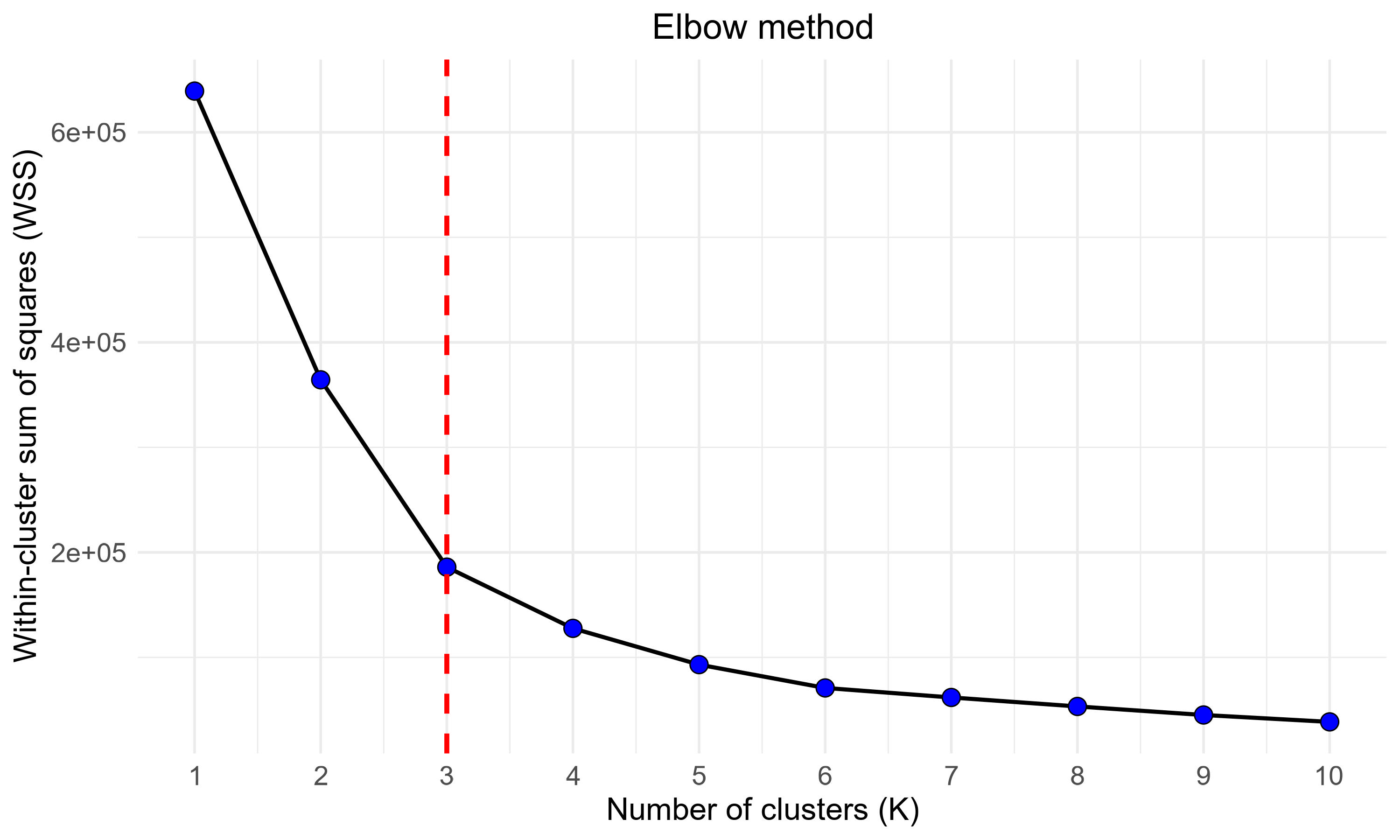

Supplement: Supplementary Figure 2 — Estimation of the optimal number of clusters using the Elbow method. [file Image2.jpg]

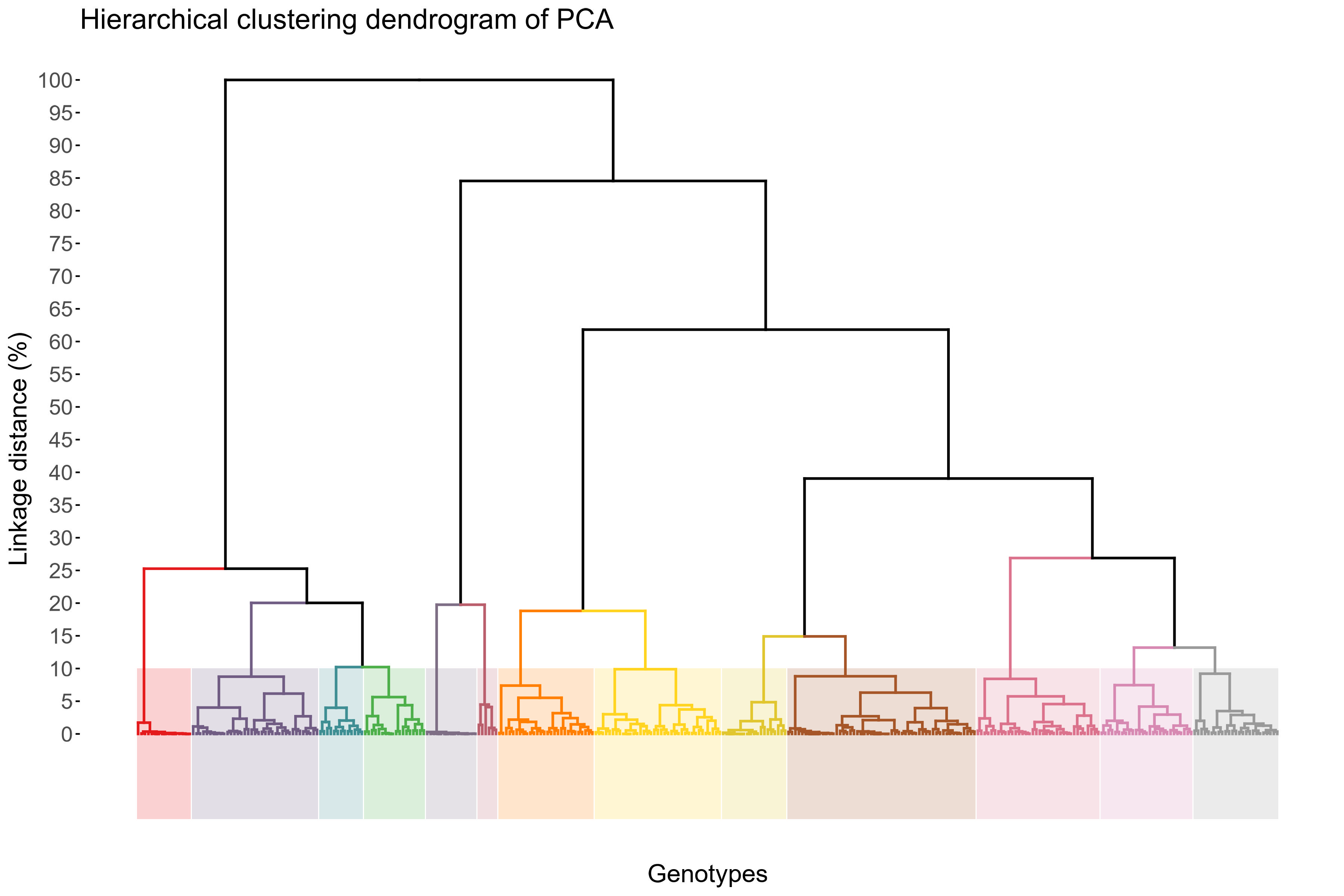

Supplement: Supplementary Figure 3 — A dendrogram of 332 genotypes using hierarchical cluster analysis of 18,417 polymorphic SNPs. [file Image3.jpg]

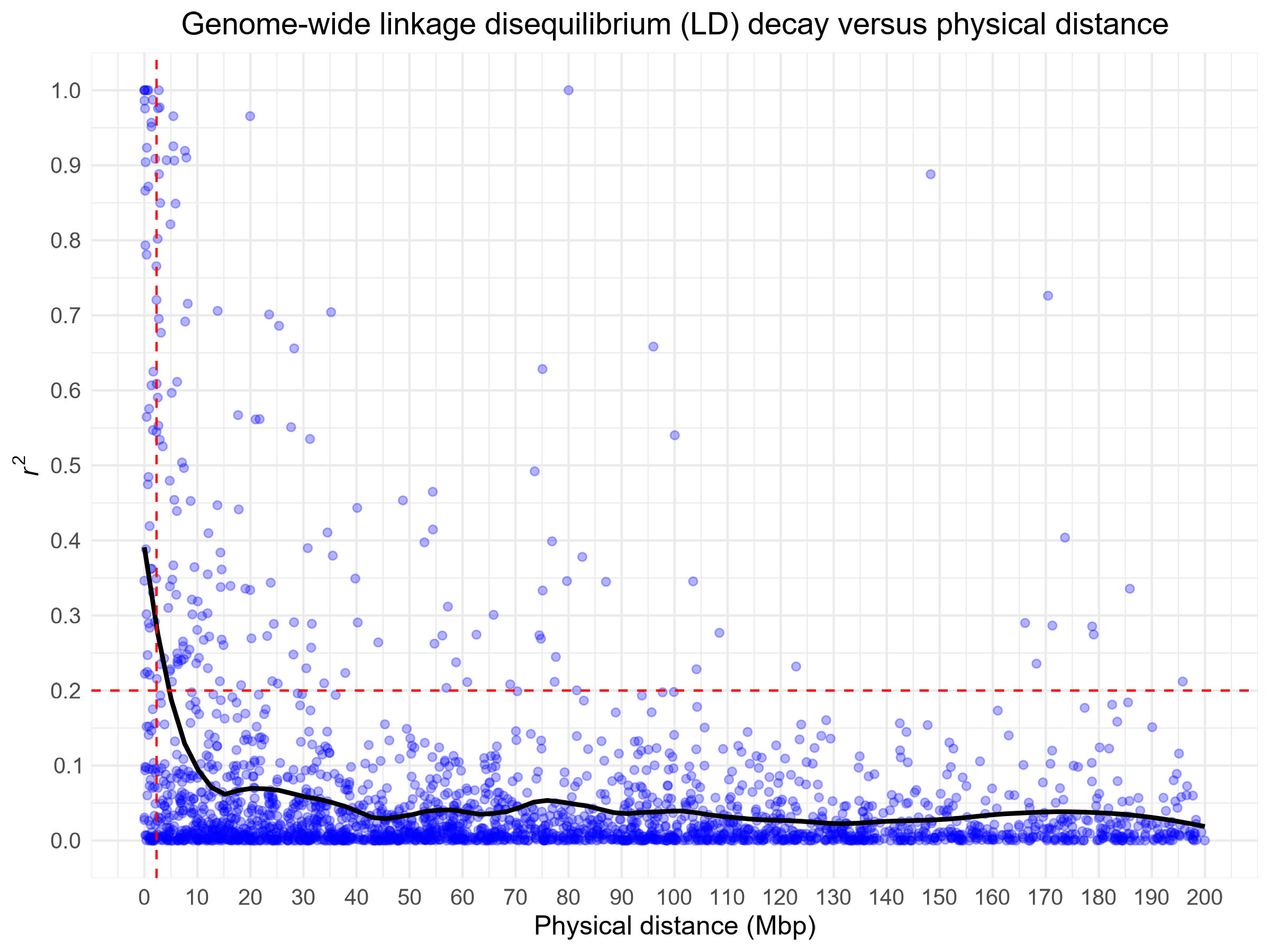

Supplement: Supplementary Figure 4 — Scatter plot of genome-wide linkage disequilibrium (LD) (r 2) versus physical distance (Mbp) for the 332 spring wheat genotypes based on 18,417 SNP markers within whole genome. The fitted locally weighted polynomial regression-based (LOESS) curve is indicated. The red dashed lines demonstrate the intersection between the critical LD value r² = 0.2 and physical distance. [file Image4.jpg]

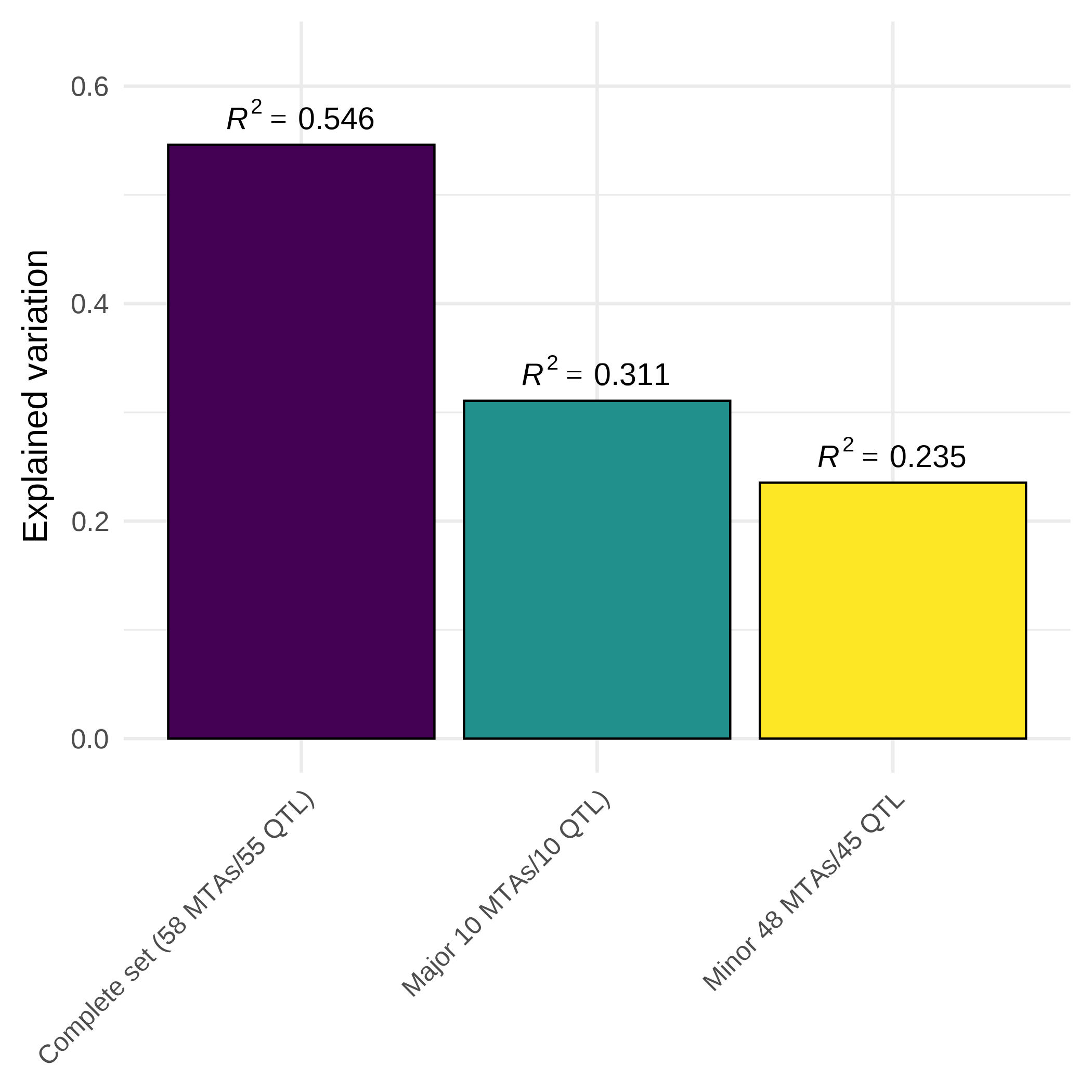

Supplement: Supplementary Figure 5 — Proportion of explained variation by three QTL groups: 55 QTL, 10 major and 45 minor in wheat resistance (BLUEs across eight trials) [file Image5.jpg]

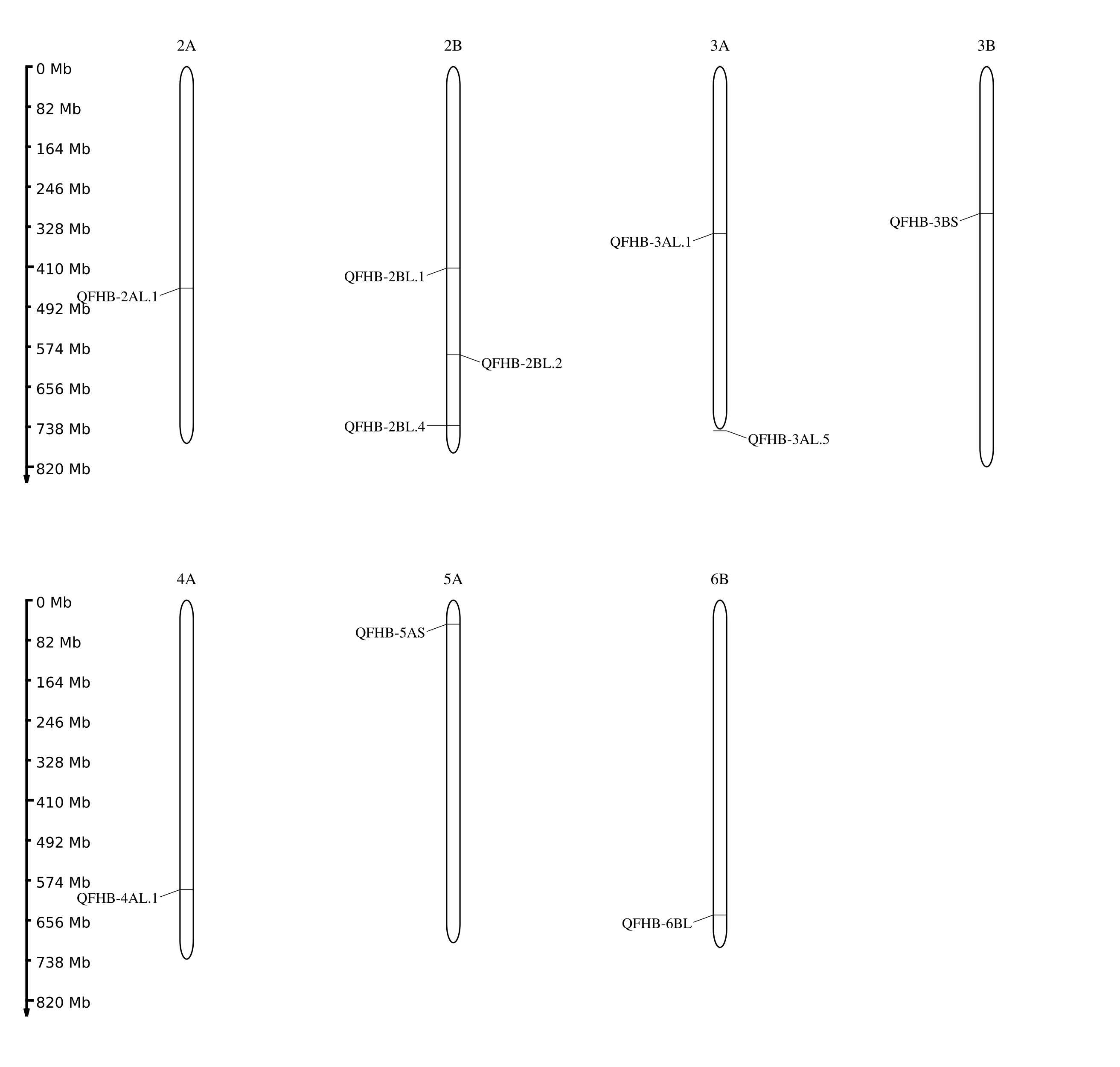

Supplement: Supplementary Figure 6 — Distribution of major QTL on wheat chromosomes based on RefSeq v1.1. [file Image6.jpg]
